# Perioperative Intravenous Antibiotic Regimens and Surgical Site Infection After Colorectal Surgery: Clinical Associations and External Evaluation of Risk Prediction Models

**DOI:** 10.3390/idr18050113

**Published:** 2026-10-06

**Authors:** Junqi Cui, Enoch Chi Ngai Lim, Zizhu Zhao, Fang Chen, Chi Eung Danforn Lim

**Affiliations:** 1 Vanke School of Public Health, Tsinghua University, Beijing 100084, China; 2 Specialist Medical Services Group, Translational Research Department, Earlwood, NSW 2206, Australia; 3 Faculty of Engineering and Information Technology, University of Technology Sydney, Ultimo, NSW 2007, Australia; 4 Data Science Institute, University of Technology Sydney, Ultimo, NSW 2007, Australia; 5 NICM(National Institute of Complementary Medicine) Health Research Institute, Western Sydney University, Westmead, NSW 2145, Australia

**Keywords:** colorectal surgery, surgical site infection, perioperative antimicrobial prophylaxis, antimicrobial stewardship, risk prediction, external evaluation

## Abstract

Background/Objectives: Surgical site infection (SSI) remains a major complication after colorectal surgery. Comparative evidence for commonly used perioperative intravenous antibiotic regimens is limited, and risk prediction models require external evaluation before clinical use. We examined regimen–SSI associations and secondarily evaluated models using routine perioperative variables to distinguish patients with and without electronically recorded postoperative SSI. Methods: We analysed 732 adults from MIMIC-IV for regimen association and model development and 133 adults from ICU-based eICU-CRD for external model evaluation. SSI was identified using an electronic phenotype. Four intravenous antibiotic exposure groups were compared using multivariable logistic regression and exploratory interaction analyses. Predictors selected using LASSO screening followed by clinical refinement informed six models, which were assessed using discrimination, calibration, and classification metrics. Results: SSI was recorded in 146/732 patients (19.9%), with no evidence of an unadjusted association across observed exposure categories (*p* = 0.947). Logistic regression achieved an external ROC-AUC of 0.858 (95% CI 0.764–0.934; PR-AUC 0.560) and detected 17/21 SSI cases, but generated 27 false positives. Positive predictive value was 38.6%, with limited calibration (expected calibration error 0.236) and a Brier score of 0.197. Conclusions: These data provide no evidence of an overall association, nor evidence of equivalent prophylactic efficacy. Heterogeneous exposures and residual confounding preclude regimen-level causal conclusions. The small ICU-based external evaluation informs higher-acuity populations, not routine elective surgery. Uncertain SSI onset, limited calibration, and false-positive burden preclude clinical implementation. Prospective multicentre studies should evaluate risk-adapted surveillance, not model-directed antibiotic selection.

## 1. Introduction

Surgical site infection (SSI) remains one of the most frequent complications following colorectal surgery [1,2,3,4,5]. Colorectal procedures expose the operative field to aerobic and anaerobic enteric organisms, creating a substantial risk of bacterial contamination [4,5]. SSI is associated with increased postoperative morbidity, prolonged hospitalisation, readmission, and substantial additional healthcare expenditure [6,7]. Prevention is therefore a major priority for patient safety and surgical quality improvement [1,2,3,8].

Perioperative antimicrobial prophylaxis is central to SSI prevention in colorectal surgery and should achieve adequate serum and tissue concentrations at incision while covering aerobic and anaerobic bowel flora [1,2,9,10,11]. The joint ASHP/IDSA/SIS/SHEA guideline includes cefazolin–metronidazole, ceftriaxone–metronidazole, cefoxitin, cefotetan, ampicillin–sulbactam, and ertapenem among recommended options [10]. Regimen selection should account for documented allergy, prior colonisation, or infection with resistant organisms, local susceptibility patterns, and antimicrobial stewardship principles, while avoiding unnecessarily broad or prolonged exposure [1,8,10,11,12]. Comparative analyses of routine-care regimens are clinically relevant, but their interpretation also depends on dose, timing, intraoperative redosing, and operative context.

Within multimodal perioperative prophylaxis, comparative studies have primarily examined the incremental benefit of adding oral antibiotics to intravenous prophylaxis [13,14,15]. Evidence directly guiding selection among intravenous agents and combinations remains limited [16,17]. A network meta-analysis of 105 randomised trials reported the strongest associations with reduced SSI risk for broad-spectrum penicillins and cephalosporin-based regimens [16]. However, many individual comparisons were based on small trials, and incomplete reporting of patient and procedural characteristics limited the assessment of clinically relevant heterogeneity [16]. Observational comparisons can therefore generate useful hypotheses but cannot establish causal superiority or equivalence among pooled antibiotic categories.

SSI risk also reflects patient- and procedure-related factors, including comorbidity, frailty, operative duration, and surgical complexity [1,4,5,18,19,20]. Risk stratification may identify patients who could benefit from targeted preventive interventions and enhanced postoperative monitoring [19,20,21,22,23]. Published colorectal SSI risk models have generally shown reduced or inconsistent performance in independent populations [24,25], while systematic reviews have identified limited external validation, infrequent calibration assessment, and substantial risk of bias [21,26]. Clinical evaluation should therefore consider discrimination, calibration, threshold-specific performance, and clinical utility before implementation [21,24,26,27,28,29].

The primary objective of this study was to evaluate associations between observed perioperative intravenous antibiotic exposure categories and SSI in adults undergoing colorectal surgery. A secondary objective was to develop and externally evaluate models using routinely available perioperative variables to distinguish patients with and without electronically recorded postoperative SSI. The association analysis describes observed prescribing patterns and SSI. The secondary modelling analysis explores a potential basis for future research on risk-adapted surveillance. Neither analysis estimates the benefit of changing an individual patient’s antibiotic.

## 2. Materials and Methods

### 2.1. Study Design

This retrospective cohort study primarily used the Medical Information Mart for Intensive Care IV (MIMIC-IV, version 3.1) database as the main database for analysis. MIMIC-IV contains deidentified hospitalisation records collected at Beth Israel Deaconess Medical Center between 2008 and 2022 [30]. The database was deidentified in accordance with Health Insurance Portability and Accountability Act requirements. Its use was approved by the institutional review boards of Beth Israel Deaconess Medical Center and the Massachusetts Institute of Technology, with the requirement for individual informed consent waived.

The eICU Collaborative Research Database (eICU-CRD, version 2.0) was also used as an external evaluation cohort. The eICU-CRD contains deidentified critical-care data collected from 208 hospitals across the United States between 2014 and 2015, providing a geographically and institutionally distinct multicentre population [31]. Because eICU-CRD contains ICU encounters, external generalisability is primarily to higher-acuity postoperative populations rather than to all patients undergoing elective colorectal surgery. The database was released under the Health Insurance Portability and Accountability Act safe-harbour provision, and individual informed consent was not required for this secondary analysis.

Adult patients who underwent colorectal resection were identified separately in the two databases. In MIMIC-IV, eligible operations were identified using International Classification of Diseases procedure codes. Duplicate procedure records and endoscopic, diagnostic, or non-resection procedures were excluded. Patients younger than 18 years or with fewer than 2 days of postoperative in-hospital observation were also excluded. To reduce inclusion of therapeutic antimicrobial treatment, patients with perforation, peritonitis, active infection, or another documented indication for treatment at the time of surgery were excluded.

Perioperative intravenous antibiotic exposure was confirmed using recorded medication administrations rather than medication orders. The index operation time was defined using the recorded operative procedure timestamp, and intravenous route was identified from the corresponding medication administration records. Eligible perioperative exposure was defined as intravenous antibiotic administration initiated within 120 min before the index operation and continued for no longer than 24 h postoperatively. Antibiotic administrations during this 24 h postoperative window were used to define the observed perioperative regimen and exposure duration.

Courses extending beyond 24 h were not classified as perioperative prophylaxis. Patients receiving such courses were excluded when therapeutic intent was documented or could not be reliably ruled out. Patients with infection present at the time of surgery were also excluded. In eICU-CRD, the same temporal exposure definition was applied using the available procedure and medication timing records. Patients without sufficient information to confirm intravenous perioperative exposure were excluded. For prediction modelling, the prediction time was defined after completion of the 24 h perioperative exposure window. Laboratory predictors were calculated as the mean of all available preoperative measurements within the 7 days preceding the operation. No postoperative laboratory measurements were included as predictors.

In eICU-CRD, adults with colorectal resection records were identified from available procedure information. Duplicate, endoscopic, diagnostic, local-excision, and other non-resection procedure records were excluded. Patients with pre-existing infection, fewer than 2 days of in-hospital observation, or insufficient evidence of perioperative intravenous antibacterial exposure were also excluded. Variables with more than 30% missing data were excluded. For conventional statistical analyses, remaining missing covariate values were handled using multiple imputation by chained equations with 20 imputed datasets. The imputation model included variables from the corresponding analytical models. Estimates were pooled using Rubin’s rules [32]. For prediction modelling, the MIMIC-IV cohort was first divided into training and internal validation cohorts in a 7:3 ratio at the patient level. Multiple imputation was performed separately within the training, internal validation, and external evaluation cohorts, with 20 imputed datasets generated for each cohort. For each model, patient-level predicted probabilities were generated separately in each of the 20 imputed datasets and averaged across imputations to obtain a single predicted probability for each patient. Model-specific thresholds were then applied to the averaged probabilities to obtain final binary classifications. Discrimination and calibration metrics were calculated from the averaged probabilities, whereas confusion matrices and threshold-dependent performance measures were calculated from the corresponding final classifications. Outcome information was not used to impute predictor variables in the internal validation or external evaluation cohorts. All laboratory predictors in eICU-CRD were restricted to measurements obtained before surgery and before the defined prediction time. Standardisation, feature selection, model fitting, and hyperparameter optimisation were performed using only the training cohort. The fitted transformations and model parameters were applied unchanged to the internal validation and external evaluation cohorts. Imputation, in contrast, was performed separately within each cohort.

**Figure 1:**
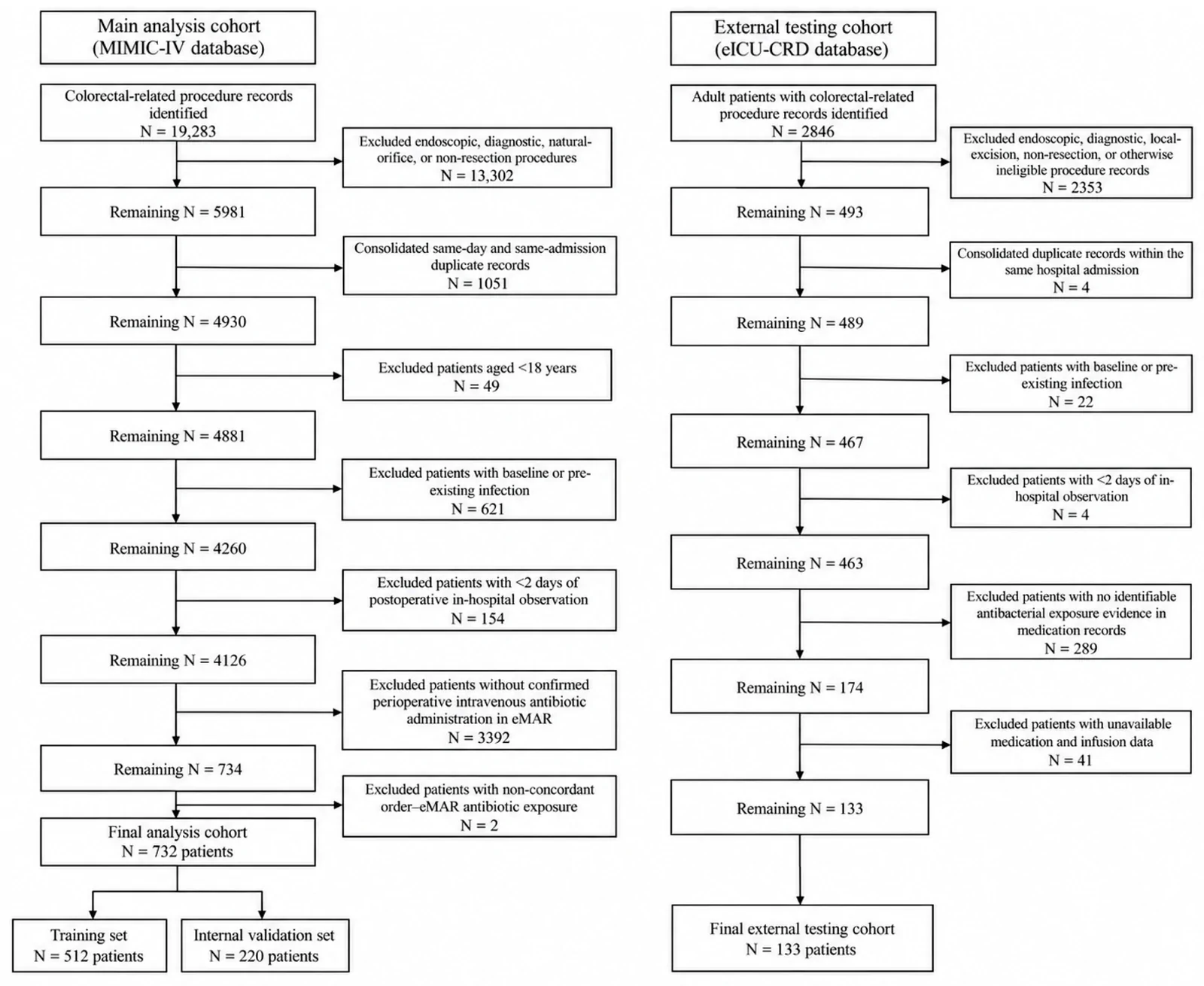
Study flowchart for the MIMIC-IV and eICU-CRD cohorts.

**Figure 2:**
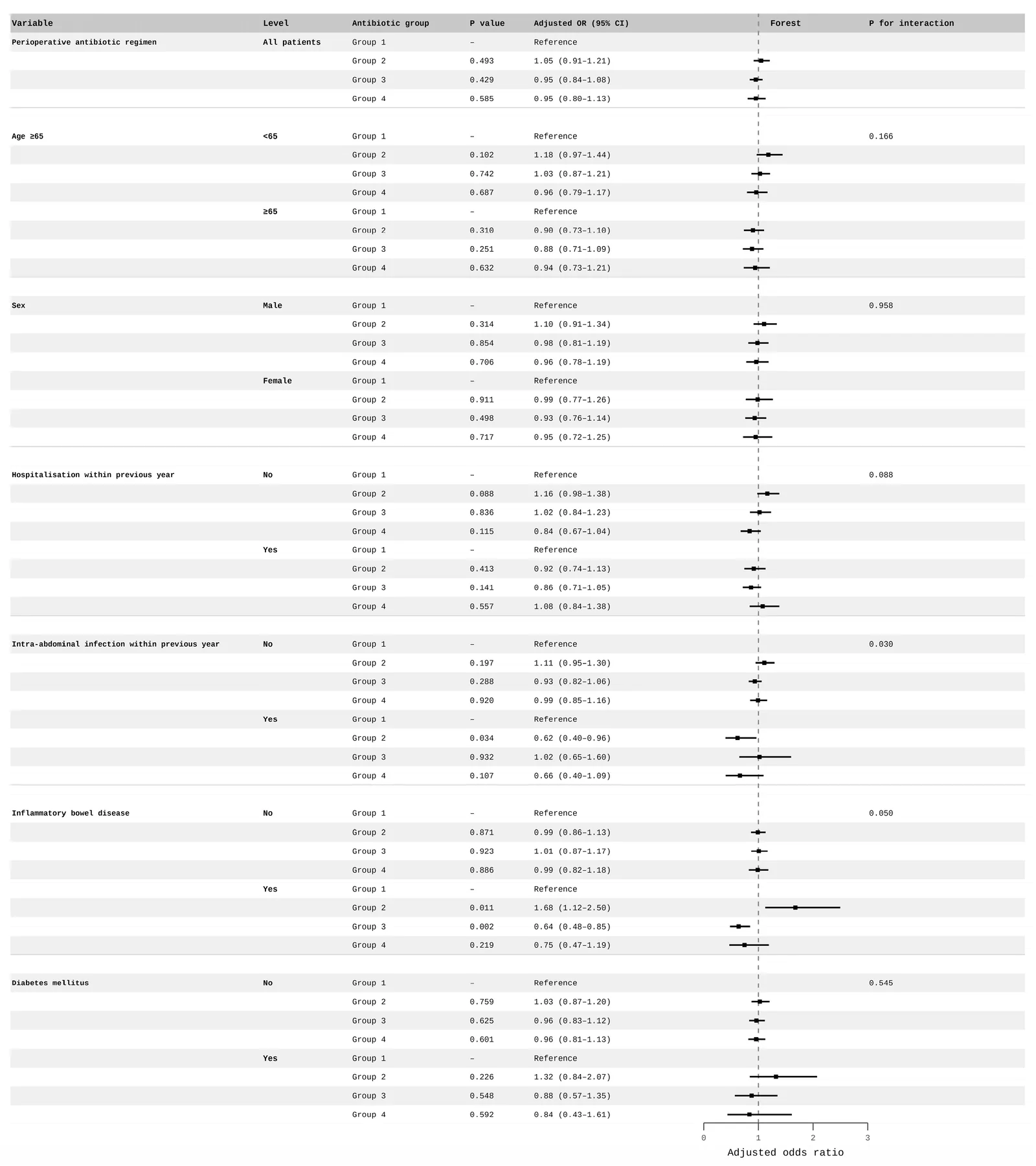
Adjusted associations between observed perioperative intravenous antibiotic groups and SSI in the overall cohort and selected exploratory subgroups.

Perioperative intravenous antibiotic exposures were classified into four mutually exclusive groups (Supplementary Table S9). These descriptive categories summarised recorded combinations by antibacterial components and coverage, rather than defining interchangeable prophylactic strategies. Group 1 comprised fluoroquinolone–metronidazole combinations and served as the reference group. Group 2 comprised cephalosporin–metronidazole combinations. Group 3 comprised broad-spectrum β-lactam regimens providing aerobic and anaerobic coverage and was dominated by piperacillin–tazobactam-containing regimens. In the MIMIC-IV cohort, piperacillin–tazobactam alone accounted for 84/206 (40.8%) patients in Group 3, with additional patients receiving piperacillin–tazobactam-containing combinations. Group 4 comprised other perioperative intravenous antibiotic regimens observed in routine practice. Assignment to the four groups was based on the recorded antibacterial components of the perioperative regimen, with each patient assigned to one mutually exclusive exposure category. Major recorded regimens and grouped residual categories are summarised in Supplementary Table S9. The patient selection process for the MIMIC-IV and eICU-CRD cohorts is summarised in Figure 1. Table 1 reports overall group sizes, SSI event counts, and crude rates; Figure 2 and Supplementary Table S10 present overall and subgroup-adjusted associations. Because Groups 3 and 4 pooled clinically heterogeneous regimens with different spectra, usual prophylactic roles, and stewardship implications, their estimates were interpreted as associations for exposure categories rather than evidence that the individual agents were clinically equivalent. A separate adjusted analysis of individual regimens was not performed because several agent-level categories contained small numbers of patients and SSI events, making further subdivision prone to sparse-cell bias and unstable estimates.

### 2.2. Outcome Definitions

The primary outcome was electronically identified postoperative SSI recorded during available follow-up, up to 30 days after the colorectal operation. The surgical site comprised anatomical structures directly incised, entered, or manipulated during the operation, including the abdominal incision, laparoscopic port sites, stoma site, where applicable, gastrointestinal operative field, anastomotic region, peritoneal cavity, abdominal cavity, and pelvis.

SSI was identified using a prespecified electronic phenotype incorporating superficial incisional, deep incisional, and organ/space infections. Qualifying evidence included an explicit SSI diagnosis; a diagnosis of postprocedural, intra-abdominal, pelvic, peritoneal, intestinal, retroperitoneal, or stoma infection supported by postoperative anatomical, imaging, or microbiological evidence; or clinically obtained microbiological evidence from an anatomically relevant operative-site specimen accompanied by other predefined evidence of infection. An isolated positive drain or fluid culture was not considered sufficient for SSI classification. The same prespecified clinical definition of SSI was used in both databases, with database-specific implementation according to the diagnosis, anatomical, microbiological, and postoperative follow-up information available in each dataset. The phenotype targeted SSI occurring within 30 days after surgery, but ascertainment was limited to qualifying postoperative events captured within the available records for each database. Patients without a qualifying recorded SSI were classified as non-SSI within the available observation period; complete 30-day surveillance was not available for all patients. In eICU-CRD, ascertainment was therefore limited to SSI identifiable from the available linked records and did not assume complete capture of 30-day readmissions. Because MIMIC-IV and eICU-CRD differ in coding structure, microbiological detail, and postoperative follow-up capture, the phenotype was implemented using the corresponding available fields in each database rather than an identical code list. The final phenotype identified 146 SSI events among 732 MIMIC-IV patients and 21 among 133 eICU-CRD patients. The prediction time was defined after the 24 h postoperative exposure window. However, SSI onset relative to this time could not be determined reliably for all electronically identified events. Model performance was therefore interpreted as retrospective discrimination of recorded SSI, not prediction of new infections arising after that time. This electronic phenotype has not been formally validated against an external reference standard or independent clinical adjudication, so formal sensitivity, specificity, and positive predictive value estimates could not be derived.

### 2.3. Association Analysis

Candidate baseline and perioperative covariates were individually evaluated for association with postoperative SSI using univariable logistic regression. Crude odds ratios (ORs), 95% confidence intervals (CIs), and two-sided *p* values were estimated within each imputed dataset and pooled using Rubin’s rules (Supplementary Table S1). Clinically relevant variables that were plausibly associated with both antibiotic regimen selection and SSI risk were prioritised as potential confounders. Two statistical criteria supported identification of additional covariates and assessment of estimate stability: univariable association with SSI (*p* < 0.05) and a change of at least 10% in any antibiotic regimen OR. The latter was assessed by adding each covariate individually to the exposure-only model. This combined clinical judgment with data-dependent screening, rather than a fully prespecified causal adjustment set.

Multivariable logistic regression was used to estimate associations between perioperative intravenous antibiotic regimens and postoperative SSI. Antibiotic regimen was forced into all six nested models, with fluoroquinolone–metronidazole combination regimens serving as the reference. Retained covariates were added cumulatively in clinically defined blocks. Adjusted ORs, 95% CIs, and two-sided *p* values were reported.

Models were compared using log-likelihood, Akaike information criterion, Bayesian information criterion, and likelihood ratio tests (Supplementary Table S3). Primary model selection considered clinically relevant confounding control, model fit, convergence, estimate stability, and clinical interpretability. AIC supported comparison of model fit and parsimony. Multicollinearity was assessed using variance inflation factors (VIFs), with values < 15 considered acceptable (Supplementary Table S2 and Table S4). Model 5 was retained as the primary adjusted model. Candidate covariates were identified using clinical relevance, univariable association with SSI, and change in antibiotic-regimen estimates as supporting criteria, rather than requiring all statistical criteria to be met simultaneously. Model 5 included age, sex, previous hospitalisation, previous intra-abdominal infection, inflammatory bowel disease, immunosuppression, diabetes mellitus, lactate, blood glucose, serum sodium, total serum calcium, chronic kidney disease, blood urea nitrogen, urinary red blood cells, urinary protein, haemoglobin, haematocrit, total carbon dioxide, and base excess. The perioperative intravenous antibiotic exposure group was forced into the model.

Subgroup analyses were performed by age, sex, previous hospitalisation, previous intra-abdominal infection, inflammatory bowel disease, and diabetes mellitus. Adjusted ORs and 95% CIs were estimated using multivariable logistic regression, with fluoroquinolone–metronidazole combinations as the reference. Effect modification was assessed using antibiotic-group-by-subgroup interaction terms added to the Model 5 adjustment set, with global interaction *p* values obtained from the corresponding joint interaction tests. Separate sensitivity analyses using alternative model specifications were performed to assess the robustness of the interaction findings. These analyses were interpreted as exploratory because of limited subgroup sizes and event counts and multiple comparisons. Interpretation emphasised the primary global interaction tests and robustness across sensitivity analyses, rather than isolated within-subgroup *p* values.

### 2.4. Risk Prediction Modelling

The MIMIC-IV cohort was randomly divided into a training cohort and an internal validation cohort at a 7:3 ratio. Candidate predictors were initially screened using least absolute shrinkage and selection operator (LASSO) logistic regression in the training cohort. Continuous variables were standardised using training-cohort parameters. The inverse regularisation parameter was selected by cross-validation, and the LASSO-screened variables were subsequently refined according to clinical relevance, routine perioperative availability, and consistency of availability across the development and external datasets.

The retained predictors were used to develop logistic regression, k-nearest neighbours, random forest, XGBoost, LightGBM, and CatBoost models. Hyperparameters were optimised in the training cohort and fixed before evaluation. Antibiotic group was among the retained predictors. Predicted probabilities were therefore conditional on the recorded exposure and did not estimate the effect of choosing a different regimen.

Discrimination was assessed using ROC-AUC and PR-AUC. All six models were evaluated in the internal validation and external evaluation cohorts. Calibration was assessed using calibration plots and expected calibration error, and the Brier score quantified overall probabilistic accuracy. Threshold-dependent classification metrics were also reported. For logistic regression, the classification threshold of 0.596 was selected by maximising Youden’s J statistic during internal model development and was fixed before external evaluation. Model-specific thresholds for the remaining models were selected using the same internal procedure and were likewise fixed before external evaluation. Predictor selection, antibiotic-group encoding, standardisation, and model hyperparameters were determined before external evaluation and were not modified on the basis of eICU-CRD results. The thresholds were statistically selected rather than clinically optimised and did not account for the different clinical consequences of missed SSI cases and false-positive alerts. SHapley Additive exPlanations (SHAP) were used to describe contributions to the logistic regression model output. In the figures, “external test” or “external testing cohort” refers to the eICU-CRD external evaluation cohort. “Validation-derived threshold” refers to a threshold selected during internal model development and fixed before external evaluation. Because all six models were compared in the external cohort, this analysis is described as an external comparative evaluation rather than definitive validation of a single prespecified model.

### 2.5. Statistical Software and Reproducibility

Statistical analyses were performed using R (version 4.6.0, Foundation for Statistical Computing, Vienna, Austria), with mice (version 3.18.0, Stef van Buuren, Karin Groothuis-Oudshoorn and contributors) for multiple imputation, stats and lmtest (version 0.9-40, Torsten Hothorn, Achim Zeileis and co-authors) for regression and model comparison, and car (version 3.1-3, John Fox, Sanford Weisberg, Brad Price and contributors) for multicollinearity assessment. Machine learning analyses were conducted in Python (version 3.12, Beaverton, OR, USA) using scikit-learn (version 1.5.2, scikit-learn developers), XGBoost (version 2.1.2, XGBoost contributors), LightGBM (version 4.5.0, Microsoft Corporation, Redmond, WA, USA), CatBoost (version 1.2.7, Yandex LLC, Moscow, Russia), and SHAP (version 0.46.0, Scott Lundberg and contributors). A random seed of 42 was used throughout to ensure reproducibility. All statistical tests were two-sided, with *p* < 0.05 considered statistically significant.

## 3. Results

### 3.1. Cohort

The MIMIC-IV main cohort included 732 unique patients, each represented by one index colorectal operation, and was divided into a training cohort (*n* = 512) and an internal validation cohort (*n* = 220). The external evaluation cohort comprised 133 patients from eICU-CRD (Supplementary Table S7). SSI was recorded in 146/732 patients (19.9%) in MIMIC-IV and 21/133 patients (15.8%) in the external cohort. Baseline characteristics were generally comparable between the training and internal validation cohorts. The external cohort was older (median age, 71.0 [62.0–82.0] vs. 62.0 [50.0–72.0] and 65.0 [53.0–71.0] years) and had higher blood glucose (152.0 [122.0–190.0] vs. 120.0 [93.0–146.2] and 123.0 [99.8–152.0] mg/dL), but lower total serum calcium (7.9 [7.5–8.4] vs. 8.7 [8.2–9.1] and 8.6 [8.2–9.0] mg/dL) and blood urea nitrogen (14.0 [9.0–18.0] vs. 17.0 [10.0–24.0] and 19.0 [12.8–25.2] mg/dL; all *p* < 0.001). Serum sodium, haemoglobin, total carbon dioxide, base excess, and lactate were comparable across cohorts (all *p* > 0.05). Because the external cohort was derived from an ICU database, its results should be interpreted primarily in relation to higher-acuity postoperative populations.

### 3.2. Antibiotic Regimen–SSI Associations

In the full MIMIC-IV cohort, SSI occurred in 146/732 patients (19.9%), and crude rates were similar across Groups 1–4 (21.0%, 20.6%, 18.9%, and 19.1%, respectively; unadjusted *p* = 0.947; Table 1). Compared with Group 1, the adjusted ORs were 1.05 (95% CI 0.91–1.21; *p* = 0.493) for Group 2; 0.95 (95% CI 0.84–1.08; *p* = 0.429) for Group 3; and 0.95 (95% CI 0.80–1.13; *p* = 0.585) for Group 4 (Figure 2 and Supplementary Table S10). These comparisons showed no evidence of association and did not establish equivalent efficacy.

No evidence of effect modification was identified for age, sex, or diabetes mellitus (Figure 2). In the primary Model 5 interaction analyses, exploratory evidence of possible heterogeneity was observed for previous intra-abdominal infection (*n* = 68; global *p* for interaction = 0.030) and inflammatory bowel disease (*n* = 88; global *p* for interaction = 0.050). Six subgroup interaction tests were examined for age, sex, previous hospitalisation, previous intra-abdominal infection, inflammatory bowel disease, and diabetes mellitus. No formal adjustment for multiple testing was applied. Table 1 provides overall regimen-specific sample sizes, SSI events, and crude rates; Figure 2 and Supplementary Table S10 present overall and subgroup-adjusted estimates. Sensitivity analyses using alternative model specifications showed weaker statistical evidence for previous intra-abdominal infection, with global interaction *p* values ranging from 0.059 to 0.100 (Supplementary Figure S1). For inflammatory bowel disease, the corresponding global interaction *p* values ranged from 0.012 to 0.042 across sensitivity analyses (Supplementary Figure S2). Given the small subgroup sizes, limited event counts, and multiple comparisons, these findings are exploratory and hypothesis-generating and do not establish regimen superiority or support subgroup-based escalation of prophylaxis.

### 3.3. Risk Prediction Model Performance

**Figure 3:**
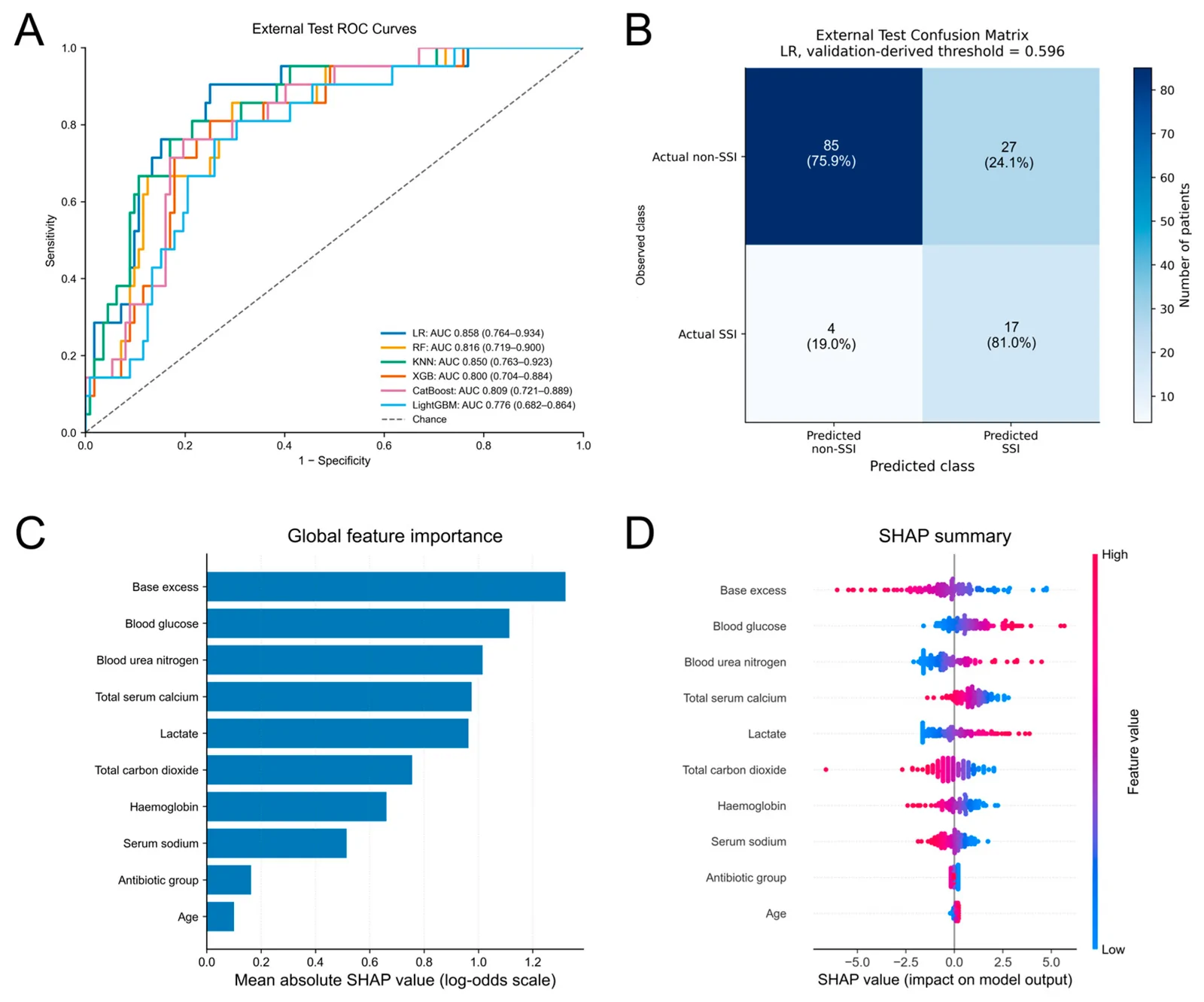
External comparative evaluation and SHAP-based interpretation of SSI risk prediction models. (**A**) Receiver operating characteristic curves for six models in the external cohort. (**B**) Confusion matrix for logistic regression at the reported threshold. (**C**,**D**) SHAP-based description of contributions to logistic regression model output.

Logistic regression achieved an ROC-AUC of 0.922 (95% CI 0.868–0.961) in the MIMIC-IV internal validation cohort and 0.858 (95% CI 0.764–0.934) in the external evaluation cohort (Figure 3A and Supplementary Figure S4). Its PR-AUC was 0.819 internally and 0.560 externally (95% CI 0.402–0.743; Supplementary Figure S5 and Figure S6). The wide confidence intervals and small external event count preclude a clear ranking of model performance. At the internally derived threshold of 0.596, which was fixed before external evaluation, logistic regression identified 17 of 21 SSI cases but generated 27 false-positive classifications, corresponding to sensitivity 81.0%, specificity 75.9%, positive predictive value 38.6% (95% CI 30.0–50.0%), and negative predictive value 95.5% (Figure 3B and Table 2). Although logistic regression had the numerically highest external ROC-AUC, calibration was limited (Brier score 0.197; expected calibration error 0.236) and was less favourable than for some competing models; it therefore did not demonstrate the best performance across all metrics. SHAP analysis of the 10 displayed predictors identified base excess as the largest contributor to model output, followed by blood glucose, blood urea nitrogen, total serum calcium, and lactate. SHAP values describe model behaviour and do not identify independent clinical risk factors, causal effects, or treatment targets (Figure 3C,D).

## 4. Discussion

The principal clinical finding was the absence of evidence for an overall association between the four observed antibiotic-exposure categories and SSI. This is not evidence of equivalence: residual confounding, confounding by indication, and heterogeneous pooled regimens preclude causal regimen comparisons. Exploratory subgroup findings cannot justify broader-spectrum prophylaxis.

The secondary modelling analysis also requires cautious interpretation. Logistic regression showed the numerically highest external ROC-AUC, yet its positive predictive value was modest, and its probability calibration was limited. The model may therefore support further research on risk-adapted surveillance, but the present results do not support routine clinical deployment, automated treatment decisions, or use of the model to select perioperative antibiotics.

### 4.1. Antibiotic Strategies and Antimicrobial Stewardship

Comparative studies have reported regimen-specific differences in SSI risk. Hendren et al. found that cefazolin–metronidazole, ciprofloxacin–metronidazole, and ertapenem were associated with lower SSI odds than alternative prophylactic antibiotics after colectomy [17]. In a randomised double-blind trial of elective colorectal surgery, the SSI rate in the modified intention-to-treat population was lower with ertapenem than with cefotetan (17.1% vs. 26.2%) [33]. A multicentre retrospective cohort study of 20,140 elective colorectal procedures similarly found that β-lactam prophylaxis was associated with lower SSI risk than non-β-lactam alternatives [34]. Nevertheless, regimen choice should remain agent-specific and account for expected organisms, documented allergy, previous colonisation or infection with resistant organisms, and local susceptibility patterns. Timing and redosing should also be considered, while unnecessarily broad or prolonged prophylaxis should be avoided [8,10,35].

The recorded SSI proportion of 19.9% is higher than in the contemporary elective cohort reported by Collins et al. (6.2% with β-lactam and 8.4% with non-β-lactam prophylaxis) [34]. Our eligibility criteria selected patients with confirmed antibiotic exposure and at least two postoperative inpatient days, rather than an unselected elective cohort. Differences in case mix and ascertainment may contribute, but the available data do not establish the explanation. This rate should not be used as a benchmark for routine elective colorectal surgery.

The subgroup analyses in the present study should be viewed as exploratory signals rather than evidence for personalised antibiotic selection. Previous intra-abdominal infection may identify patients with distinct microbiological histories, but the study did not consistently capture prior isolates, resistance patterns, or concordance between previous organisms and the perioperative regimen. In inflammatory bowel disease, intestinal dysbiosis, immunosuppressive treatment, nutritional status, disease phenotype, and operative complexity may all influence SSI risk [12,36,37]. These factors were not fully measured and may explain part of the observed associations. Confirmation requires adequately powered, prospectively specified comparisons with detailed agent-level, microbiological, and operative data.

From an antimicrobial stewardship perspective, the findings should not prompt routine escalation to piperacillin–tazobactam, carbapenems, or other broader-spectrum agents. Group 3 was dominated by piperacillin–tazobactam-containing regimens but also included other broad-spectrum β-lactam regimens with different spectra, usual prophylactic roles, and ecological consequences. Confounding by indication is also plausible because broader-spectrum agents may have been selected for patients perceived to be at greater risk of infection. The null association cannot distinguish genuinely similar effects from confounding that masks benefit or harm.

Clinically, established prophylaxis guidance and local stewardship policy should remain the basis of practice. Prior microbiology, recent antimicrobial exposure, allergy, immunosuppression, nutritional status, local resistance patterns, and operative context should inform individualised assessment [8,10,35]. Neither subgroup membership nor a predicted SSI probability should independently determine antibiotic escalation. Risk-adapted postoperative surveillance remains a proposed use for prospective investigation, not an intervention evaluated here.

### 4.2. Model Evaluation and Clinical Utility

Logistic regression achieved an ROC-AUC of 0.922 in the internal validation cohort and 0.858 in the external evaluation cohort, indicating good discrimination of recorded SSI. Discrimination alone does not establish transportability or clinical usefulness. The external evaluation cohort included only 133 patients and 21 SSI events. It was ICU-based and differed from the development cohort in several characteristics. These results do not establish transportability to routine elective colorectal surgery. The logistic regression Brier score and expected calibration error also indicated limitations in probabilistic accuracy and calibration. Recalibration and further evaluation are required before clinical use.

At the reported threshold, logistic regression produced 17 true-positive and 27 false-positive classifications and missed 4 SSI cases. Thus, most positive classifications were false positives. A clinically appropriate threshold would depend on the intended application. Lower thresholds would favour sensitivity and reduce missed cases, at the cost of more false-positive alerts if the model were deployed. This trade-off might be acceptable for a low-burden surveillance intervention, but clinical benefit cannot be inferred from accuracy metrics alone. Net clinical benefit was not demonstrated. Before clinical use, the model requires prospective multicentre evaluation and recalibration where necessary. Such studies should determine whether risk-stratified surveillance improves timely SSI recognition without excessive workload, unnecessary investigations, or antimicrobial exposure.

### 4.3. Interpretation of Model Predictors

SHAP analysis identified base excess as the largest contributor to model output, followed by blood glucose, blood urea nitrogen, total serum calcium, and lactate. These findings describe model behaviour, not independent clinical risk factors, biological causation, or modifiable treatment targets. Greater postoperative reductions in base excess have been observed among patients who developed intra-abdominal infection after pancreatic resection [38], while elevated preoperative HbA1c has been associated with SSI after elective colon cancer surgery [18,39]. The clinical meaning of these features should be interpreted in the context of their preoperative measurement window, with laboratory predictors calculated from measurements obtained within the 7 days preceding surgery.

Higher postoperative lactate has been associated with intra-abdominal infection after pancreatic resection and higher intraoperative lactate with SSI after bowel resection for Crohn’s disease [38,40]. Lactate may therefore reflect perioperative perfusion and metabolic stress. Haemoglobin may reflect oxygen-carrying capacity and underlying anaemia, which has been associated with incisional SSI after elective colorectal cancer surgery [41]. These associations from other studies provide clinical context. They do not establish the same relationships for preoperative measurements or show that changing an individual predictor would reduce SSI risk.

### 4.4. Limitations and Future Directions

This study has several limitations. First, residual confounding and confounding by indication substantially limit the primary association analysis. A clinically prespecified causal framework would have been preferable to partly data-driven covariate selection. Prognostic importance and improved model fit do not establish adequate control of confounding. Second, the databases did not consistently capture antibiotic dose, intraoperative redosing, oral prophylaxis, mechanical bowel preparation, β-lactam allergy, previous resistant-organism status, operative approach, procedure duration, wound classification, or operative complexity. The exposure groups, particularly Groups 3 and 4, were heterogeneous and should not be interpreted as comparisons of clinically equivalent individual agents. Third, SSI ascertainment relied on an electronic phenotype and available hospital records and may have missed infections diagnosed outside the contributing health system or otherwise differed from formal prospective surveillance. Observation time, readmission capture, and microbiology availability may affect ascertainment differently between cohorts; the direction of resulting bias is uncertain. In a separate caesarean cohort, most SSIs were detected after discharge, illustrating the importance of post-discharge ascertainment rather than providing a colorectal benchmark [42]. Fourth, subgroup analyses involved small counts of patients and events and multiple comparisons, limiting precision and increasing the likelihood of chance findings. Fifth, the external evaluation cohort was small and ICU-based. It was used to compare six models rather than to provide definitive validation of a single prespecified model. SSI onset could not always be established relative to the 24 h prediction time, and some infections may already have been present. The analysis, therefore, assessed retrospective discrimination of recorded SSI, not prediction of new infections arising after that time. Sixth, despite good discrimination, logistic regression showed limited external calibration and modest positive predictive value, constraining its current clinical utility. Finally, no model underwent prospective workflow or clinical impact testing.

Future multicentre studies should prospectively collect agent-level prophylaxis data, timing and redosing, oral prophylaxis and bowel preparation, allergy and resistant-organism history, operative characteristics, microbiology, and standardised 30-day SSI outcomes. Subgroup hypotheses should be prespecified and adequately powered. Risk prediction models should be prospectively evaluated as adjuncts to postoperative surveillance rather than as tools for antibiotic selection, with particular attention to calibration, false-positive burden, and clinical workflow.

## 5. Conclusions

In this retrospective cohort, no evidence of an overall association was identified between the four observed perioperative intravenous antibiotic categories and SSI; this does not establish equivalent efficacy. The results do not justify broader prophylaxis or model-directed antibiotic selection. Logistic regression showed good discrimination of recorded SSI, but uncertain SSI onset, limited calibration, modest positive predictive value, and a small ICU-based external cohort preclude clinical implementation or extrapolation to routine elective surgery. Prospective multicentre evaluation, recalibration, and clinical utility assessment are required before considering risk-adapted postoperative surveillance.

## Figures and Tables

**Table 1:** Baseline characteristics of the MIMIC-IV main cohort by observed perioperative intravenous antibiotic group.

Variable	Overall (N = 732)	Group 1 (N = 210)	Group 2 (N = 180)	Group 3 (N = 206)	Group 4 (N = 136)	*p* Value
Patient demographics						
Age, years	60.77 ± 15.61	61.51 ± 15.31	61.22 ± 15.51	60.00 ± 15.90	60.22 ± 15.88	0.729
Sex = Female	405 (55.3%)	120 (57.1%)	100 (55.6%)	110 (53.4%)	75 (55.1%)	0.897
Sex = Male	327 (44.7%)	90 (42.9%)	80 (44.4%)	96 (46.6%)	61 (44.9%)	
Healthcare utilisation and perioperative characteristics						
Prior hospitalisation within 1 year = No	392 (53.6%)	117 (55.7%)	92 (51.1%)	118 (57.3%)	65 (47.8%)	0.285
Prior hospitalisation within 1 year = Yes	340 (46.4%)	93 (44.3%)	88 (48.9%)	88 (42.7%)	71 (52.2%)	
Clinical history and comorbidities						
Prior intra-abdominal infection within 1 year = No	664 (90.7%)	195 (92.9%)	162 (90.0%)	181 (87.9%)	126 (92.6%)	0.279
Prior intra-abdominal infection within 1 year = Yes	68 (9.3%)	15 (7.1%)	18 (10.0%)	25 (12.1%)	10 (7.4%)	
Inflammatory bowel disease = No	644 (88.0%)	188 (89.5%)	155 (86.1%)	179 (86.9%)	122 (89.7%)	0.641
Inflammatory bowel disease = Yes	88 (12.0%)	22 (10.5%)	25 (13.9%)	27 (13.1%)	14 (10.3%)	
Diabetes mellitus = No	668 (91.3%)	191 (91.0%)	163 (90.6%)	185 (89.8%)	129 (94.9%)	0.41
Diabetes mellitus = Yes	64 (8.7%)	19 (9.0%)	17 (9.4%)	21 (10.2%)	7 (5.1%)	
Chronic kidney disease = No	678 (92.6%)	196 (93.3%)	168 (93.3%)	187 (90.8%)	127 (93.4%)	0.698
Chronic kidney disease = Yes	54 (7.4%)	14 (6.7%)	12 (6.7%)	19 (9.2%)	9 (6.6%)	
Immunosuppression = No	695 (94.9%)	201 (95.7%)	170 (94.4%)	194 (94.2%)	130 (95.6%)	0.867
Immunosuppression = Yes	37 (5.1%)	9 (4.3%)	10 (5.6%)	12 (5.8%)	6 (4.4%)	
Malignancy = No	598 (81.7%)	170 (81.0%)	152 (84.4%)	165 (80.1%)	111 (81.6%)	0.72
Malignancy = Yes	134 (18.3%)	40 (19.0%)	28 (15.6%)	41 (19.9%)	25 (18.4%)	
Malnutrition within 1 year = No	685 (93.6%)	198 (94.3%)	162 (90.0%)	197 (95.6%)	128 (94.1%)	0.137
Malnutrition within 1 year = Yes	47 (6.4%)	12 (5.7%)	18 (10.0%)	9 (4.4%)	8 (5.9%)	
Tobacco use within 1 year = No	584 (79.8%)	178 (84.8%)	135 (75.0%)	160 (77.7%)	111 (81.6%)	0.085
Tobacco use within 1 year = Yes	148 (20.2%)	32 (15.2%)	45 (25.0%)	46 (22.3%)	25 (18.4%)	
Haematological profile						
Haematocrit, %	33.50 ± 6.51	34.09 ± 6.66	33.04 ± 6.87	33.44 ± 6.44	33.31 ± 5.87	0.429
Haemoglobin, g/dL	10.85 ± 2.24	10.96 ± 2.21	10.74 ± 2.26	10.66 ± 2.28	11.09 ± 2.22	0.275
Red blood cell count, 10^12^/L	3.68 ± 0.79	3.73 ± 0.78	3.70 ± 0.78	3.65 ± 0.82	3.64 ± 0.76	0.607
White blood cell count, 10^9^/L	10.19 ± 4.83	9.90 ± 4.74	9.79 ± 4.82	10.40 ± 4.70	10.84 ± 5.15	0.185
Platelet count, 10^9^/L	273.45 ± 123.96	280.48 ± 123.39	285.30 ± 127.63	252.60 ± 121.44	278.47 ± 121.19	0.039
Renal function and glucose metabolism						
Serum creatinine, mg/dL	1.27 ± 0.69	1.26 ± 0.73	1.14 ± 0.64	1.40 ± 0.72	1.25 ± 0.65	0.004
Blood urea nitrogen, mg/dL	18.41 ± 10.42	17.73 ± 10.03	16.83 ± 10.07	20.34 ± 10.60	18.63 ± 10.80	0.007
Blood glucose, mg/dL	122.55 ± 39.60	121.89 ± 39.02	119.50 ± 39.00	118.32 ± 38.12	134.04 ± 41.68	0.002
Electrolyte and mineral profile						
Serum sodium, mmol/L	138.74 ± 3.66	138.69 ± 3.76	138.90 ± 3.63	138.73 ± 3.39	138.63 ± 3.97	0.925
Serum potassium, mmol/L	4.11 ± 0.54	4.10 ± 0.53	4.09 ± 0.56	4.19 ± 0.54	4.04 ± 0.54	0.062
Serum chloride, mmol/L	102.48 ± 4.92	101.94 ± 5.20	103.45 ± 4.53	101.92 ± 4.64	102.90 ± 5.17	0.004
Total serum calcium, mg/dL	8.66 ± 0.68	8.73 ± 0.67	8.68 ± 0.68	8.63 ± 0.71	8.56 ± 0.63	0.155
Serum magnesium, mg/dL	2.04 ± 0.30	2.03 ± 0.29	2.07 ± 0.33	2.04 ± 0.27	2.03 ± 0.30	0.664
Serum phosphate, mg/dL	3.48 ± 0.86	3.53 ± 0.79	3.50 ± 0.82	3.55 ± 0.92	3.28 ± 0.93	0.026
Acid–base and metabolic profile						
Total carbon dioxide, mmol/L	22.18 ± 4.52	22.21 ± 4.54	22.24 ± 4.74	21.89 ± 4.36	22.46 ± 4.46	0.714
Bicarbonate, mmol/L	24.37 ± 3.87	24.72 ± 3.94	24.11 ± 4.05	24.21 ± 3.94	24.41 ± 3.39	0.416
Lactate, mmol/L	2.46 ± 1.37	2.23 ± 1.25	2.31 ± 1.28	2.57 ± 1.37	2.83 ± 1.59	<0.001
Base excess, mmol/L	−3.10 ± 6.92	−3.27 ± 6.80	−3.96 ± 6.82	−2.57 ± 7.08	−2.51 ± 6.93	0.161
Urinalysis						
Urinary red blood cells, cells/HPF	19.00 (9.00–35.00)	21.00 (9.25–37.75)	20.00 (10.00–35.00)	16.00 (7.25–31.75)	20.00 (10.75–33.00)	0.209
Urinary protein, mg/dL	125.00 (63.50–212.50)	119.00 (54.25–206.50)	114.50 (48.50–197.50)	145.00 (75.25–227.00)	121.50 (71.50–215.75)	0.121
Postoperative outcomes						
Postoperative SSI = No	586 (80.1%)	166 (79.0%)	143 (79.4%)	167 (81.1%)	110 (80.9%)	0.947
Postoperative SSI = Yes	146 (19.9%)	44 (21.0%)	37 (20.6%)	39 (18.9%)	26 (19.1%)	

Table note: Values are mean ± standard deviation, median (interquartile range), or *n* (%), as presented. *p* values are descriptive unadjusted comparisons across observed regimen groups and should not be interpreted as evidence of causal balance. Abbreviations: HPF, high-power field; SSI, surgical site infection.

**Table 2:** Threshold-dependent performance and calibration metrics for six risk prediction models in the external evaluation cohort.

Model	Accuracy (95% CI)	Sensitivity (95% CI)	Specificity (95% CI)	PPV (95% CI)	F1 Score (95% CI)	Brier Score	ECE
Logistic regression	0.767 (0.692–0.842)	0.810 (0.619–0.952)	0.759 (0.679–0.839)	0.386 (0.300–0.500)	0.523 (0.418–0.633)	0.197	0.236
Random forest	0.805 (0.737–0.872)	0.667 (0.476–0.857)	0.830 (0.759–0.902)	0.424 (0.303–0.571)	0.519 (0.370–0.655)	0.139	0.177
KNN	0.684 (0.602–0.759)	0.857 (0.667–1.000)	0.652 (0.562–0.741)	0.316 (0.254–0.389)	0.462 (0.376–0.548)	0.107	0.067
XGBoost	0.805 (0.737–0.872)	0.714 (0.524–0.905)	0.821 (0.750–0.893)	0.429 (0.317–0.571)	0.536 (0.400–0.667)	0.135	0.104
CatBoost	0.729 (0.654–0.805)	0.762 (0.571–0.905)	0.723 (0.643–0.804)	0.340 (0.255–0.436)	0.471 (0.358–0.585)	0.137	0.111
LightGBM	0.722 (0.647–0.797)	0.762 (0.571–0.905)	0.714 (0.634–0.795)	0.333 (0.255–0.422)	0.464 (0.356–0.567)	0.148	0.111

Table note: Accuracy, sensitivity, specificity, PPV, and F1 score are reported with 95% confidence intervals estimated using 2000 patient-level stratified bootstrap resamples. PPV, positive predictive value; ECE, expected calibration error. Lower Brier scores indicate better overall probabilistic accuracy, whereas lower ECE indicates closer agreement between predicted probabilities and observed outcomes. Logistic regression had the numerically highest external ROC-AUC and PR-AUC point estimates but did not have the lowest Brier score or ECE, nor the highest F1 score.

## Data Availability

The datasets analysed in this study are available through PhysioNet. MIMIC-IV (version 3.1) and eICU-CRD (version 2.0) are accessible to credentialed users who complete the required training and accept the relevant data use agreements. Both datasets were deidentified before release. No additional unpublished dataset was generated by the authors.

## Supplementary Materials

The following supporting information can be downloaded at https://www.mdpi.com/article/10.3390/idr18050113/s1, Figure S1: Sensitivity analyses of effect modification by previous intra-abdominal infection on the association between perioperative antibiotic regimen and surgical site infection; Figure S2: Sensitivity analyses of effect modification by inflammatory bowel disease on the association between perioperative antibiotic regimen and surgical site infection; Figure S3: LASSO coefficient paths and selection of the optimal regularisation parameter; Figure S4: Receiver operating characteristic curves of six models in the MIMIC-IV internal validation cohort; Figure S5: Precision–recall curves of six models in the MIMIC-IV internal validation cohort; Figure S6: Precision–recall curves of six models in the independent external evaluation cohort; Figure S7: Calibration curves of six models in the MIMIC-IV internal validation cohort; Figure S8: Calibration curves of six models in the independent external evaluation cohort; Figure S9: Confusion matrix of the logistic regression model in the MIMIC-IV internal validation cohort at the validation-derived threshold; Table S1: Univariable associations between candidate covariates and postoperative surgical site infection; Table S2: Multicollinearity assessment of candidate covariates; Table S3: Comparison of sequential multivariable logistic regression models for postoperative surgical site infection; Table S4: Multicollinearity assessment of covariates; Table S5: Variable dictionary for machine learning model development; Table S6: Model parameter table; Table S7: Baseline characteristics of the training, internal validation, and external evaluation cohorts; Table S8: Performance of six machine learning models in the internal validation cohort; Table S9: Distribution of major recorded perioperative intravenous regimens and grouped residual categories within each antibiotic exposure group in the MIMIC-IV cohort; Table S10: Overall and subgroup-specific associations between perioperative intravenous antibiotic regimens and surgical site infection after colorectal surgery; Table S11: Adjusted associations between perioperative intravenous antibiotic regimen groups and postoperative surgical site infection in model 5.

## Abbreviations

The following abbreviations are used in this manuscript:

AI	Artificial intelligence
AIC	Akaike information criterion
ASHP	American Society of Health-System Pharmacists
CatBoost	Categorical Boosting
CI	Confidence interval
ECE	Expected calibration error
eICU-CRD	eICU Collaborative Research Database
HbA1c	Glycated haemoglobin (haemoglobin A1c)
HPF	High-power field
ICU	Intensive care unit
IDSA	Infectious Diseases Society of America
KNN	k-nearest neighbours
LASSO	Least absolute shrinkage and selection operator
LightGBM	Light Gradient Boosting Machine
LR	Logistic regression
MIMIC-IV	Medical Information Mart for Intensive Care IV
OR	Odds ratio
PPV	Positive predictive value
PR-AUC	Area under the precision–recall curve
RF	Random forest
ROC-AUC	Area under the receiver operating characteristic curve
SHAP	SHapley Additive exPlanations
SHEA	Society for Healthcare Epidemiology of America
SIS	Surgical Infection Society
SSI	Surgical site infection
VIF	Variance inflation factor
XGB	XGBoost/Extreme Gradient Boosting
XGBoost	Extreme Gradient Boosting

## References

[1] Shogan B.D., Vogel J.D., Davis B.R., Keller D.S., Ayscue J.M., Goldstein L.E., Feingold D.L., Lightner A.L., Paquette I.M. (2024). The American Society of Colon and Rectal Surgeons clinical practice guidelines for preventing surgical site infection. Dis. Colon Rectum.

[2] Berríos-Torres S.I., Umscheid C.A., Bratzler D.W., Leas B., Stone E.C., Kelz R.R., Reinke C.E., Morgan S., Solomkin J.S., Mazuski J.E. (2017). Centers for Disease Control and Prevention guideline for the prevention of surgical site infection, 2017. JAMA Surg..

[3] Allegranzi B., Bischoff P., de Jonge S., Kubilay N.Z., Zayed B., Gomes S.M., Abbas M., Atema J.J., Gans S., van Rijen M. (2016). New WHO recommendations on preoperative measures for surgical site infection prevention: An evidence-based global perspective. Lancet Infect. Dis..

[4] Paulson E.C., Thompson E., Mahmoud N. (2017). Surgical site infection and colorectal surgical procedures: A prospective analysis of risk factors. Surg. Infect..

[5] Hou T.Y., Gan H.Q., Zhou J.F., Gong Y.J., Li L.Y., Zhang X.Q., Meng Y., Chen J.R., Liu W.J., Ye L. (2020). Incidence of and risk factors for surgical site infection after colorectal surgery: A multiple-center prospective study of 3,663 consecutive patients in China. Int. J. Infect. Dis..

[6] Turner M.C., Migaly J. (2019). Surgical site infection: The clinical and economic impact. Clin. Colon Rectal Surg..

[7] Mahmoud N.N., Turpin R.S., Yang G., Saunders W.B. (2009). Impact of surgical site infections on length of stay and costs in selected colorectal procedures. Surg. Infect..

[8] Calderwood M.S., Anderson D.J., Bratzler D.W., Dellinger E.P., Garcia-Houchins S., Maragakis L.L., Nyquist A.C., Perkins K.M., Preas M.A., Saiman L. (2023). Strategies to prevent surgical site infections in acute-care hospitals: 2022 update. Infect. Control Hosp. Epidemiol..

[9] Nelson R.L., Gladman E., Barbateskovic M. (2014). Antimicrobial prophylaxis for colorectal surgery. Cochrane Database Syst. Rev..

[10] Bratzler D.W., Dellinger E.P., Olsen K.M., Perl T.M., Auwaerter P.G., Bolon M.K., Fish D.N., Napolitano L.M., Sawyer R.G., Slain D. (2013). Clinical practice guidelines for antimicrobial prophylaxis in surgery. Am. J. Health Syst. Pharm..

[11] Seidelman J.L., Mantyh C.R., Anderson D.J. (2023). Surgical site infection prevention: A review. JAMA.

[12] Lim E.C.N., Lim C.E.D. (2025). Microbiome-targeted therapies in gastrointestinal diseases: Clinical evidence and emerging innovations. Acta Microbiol. Hell..

[13] Toh J.W.T., Phan K., Hitos K., Pathma-Nathan N., El-Khoury T., Richardson A.J., Morgan G., Engel A., Ctercteko G. (2018). Association of mechanical bowel preparation and oral antibiotics before elective colorectal surgery with surgical site infection: A network meta-analysis. JAMA Netw. Open.

[14] McSorley S.T., Steele C.W., McMahon A.J. (2018). Meta-analysis of oral antibiotics, in combination with preoperative intravenous antibiotics and mechanical bowel preparation the day before surgery, compared with intravenous antibiotics and mechanical bowel preparation alone to reduce surgical-site infections in elective colorectal surgery. BJS Open.

[15] Rollins K.E., Javanmard-Emamghissi H., Acheson A.G., Lobo D.N. (2019). The role of oral antibiotic preparation in elective colorectal surgery: A meta-analysis. Ann. Surg..

[16] Motaghi S., Karam S.G., Mulazzani F., Mirzayeh Fashami F., Buchan T.A., Ibrahim S., Langeroodi S.M.F., Khademioore S., Couban R.J., Mbuagbaw L. (2026). Antibiotic prophylaxis strategies and surgical site infections in colorectal surgery: A systematic review and network meta-analysis. JAMA Netw. Open.

[17] Hendren S., Fritze D., Banerjee M., Kubus J., Cleary R.K., Englesbe M.J., Campbell D.A. (2013). Antibiotic choice is independently associated with risk of surgical site infection after colectomy: A population-based cohort study. Ann. Surg..

[18] Korol E., Johnston K., Waser N., Sifakis F., Jafri H.S., Lo M., Kyaw M.H. (2013). A systematic review of risk factors associated with surgical site infections among surgical patients. PLoS ONE.

[19] Gervaz P., Bandiera-Clerc C., Buchs N.C., Eisenring M.C., Troillet N., Perneger T., Harbarth S. (2012). Scoring system to predict the risk of surgical-site infection after colorectal resection. Br. J. Surg..

[20] Amri R., Dinaux A.M., Kunitake H., Bordeianou L.G., Berger D.L. (2017). Risk stratification for surgical site infections in colon cancer. JAMA Surg..

[21] McLean K.A., Goel T., Lawday S., Riad A., Simoes J., Knight S.R., Ghosh D., Glasbey J.C., Bhangu A., Harrison E.M. (2023). Prognostic models for surgical-site infection in gastrointestinal surgery: Systematic review. Br. J. Surg..

[22] Cui J., Li W., Lim E.C.N., Wu X., Lim C.E.D. (2026). Stratified causal inference for intensive care unit risk prediction: Informatics-based modeling of anesthetic drug combinations. JMIR Form. Res..

[23] Cui J., Lim E.C.N., Wu X., Lim C.E.D. (2025). Phenylephrine’s heterogeneous renal effects in non-cardiac surgery: A causal machine learning study. Cureus J. Comput Sci..

[24] Bergquist J.R., Thiels C.A., Etzioni D.A., Habermann E.B., Cima R.R. (2016). Failure of colorectal surgical site infection predictive models applied to an independent dataset: Do they add value or just confusion?. J. Am. Coll. Surg..

[25] Grant R., Aupee M., Buchs N.C., Cooper K., Eisenring M.C., Lamagni T., Ris F., Tanguy J., Troillet N., Harbarth S. (2019). Performance of surgical site infection risk prediction models in colorectal surgery: External validity assessment from three European national surveillance networks. Infect. Control Hosp. Epidemiol..

[26] van Boekel A.M., van der Meijden S.L., Arbous S.M., Nelissen R.G.H.H., Veldkamp K.E., Nieswaag E.B., Jochems K.F.T., Holtz J., Veenstra A.V.I., Reijman J. (2024). Systematic evaluation of machine learning models for postoperative surgical site infection prediction. PLoS ONE.

[27] Cui J., He Z., Lim E.C.N., Lim C.E.D. (2026). Precision vs recall in surgical AI: Performance and limitations of tabular foundation model for perioperative classification and risk prediction. Digit. Health.

[28] Lim E.C.N., Lim C.E.D. (2026). Machine learning in perioperative medicine: A comparative review of predictive, causal, and foundation model approaches in surgical data science. AI Clin. Med..

[29] Lim E.C.N., Lim C.E.D. (2026). Responsible use of AI in healthcare: An Australian perspective on promise, perils, and professional duties. AI Ethics.

[30] Johnson A.E.W., Bulgarelli L., Shen L., Gayles A., Shammout A., Horng S., Pollard T.J., Hao S., Moody B., Gow B. (2023). MIMIC-IV, a freely accessible electronic health record dataset. Sci. Data.

[31] Pollard T.J., Johnson A.E.W., Raffa J.D., Celi L.A., Mark R.G., Badawi O. (2018). The eICU Collaborative Research Database, a freely available multi-center database for critical care research. Sci. Data.

[32] van Buuren S., Groothuis-Oudshoorn K. (2011). Mice: Multivariate imputation by chained equations in R. J. Stat. Softw..

[33] Itani K.M.F., Wilson S.E., Awad S.S., Jensen E.H., Finn T.S., Abramson M.A. (2006). Ertapenem versus cefotetan prophylaxis in elective colorectal surgery. N. Engl. J. Med..

[34] Collins C.D., Hartsfield E., Cleary R.K., Veve M.P., Brockhaus K.K. (2026). β-Lactam vs non–β-lactam prophylaxis in elective colorectal surgery. JAMA Netw. Open.

[35] Righi E., Mutters N.T., Guirao X., del Toro M.D., Eckmann C., Friedrich A.W., Giannella M., Kluytmans J., Presterl E., Christaki E. (2023). ESCMID/EUCIC clinical practice guidelines on perioperative antibiotic prophylaxis in patients colonized by multidrug-resistant Gram-negative bacteria before surgery. Clin. Microbiol. Infect..

[36] Lim E.C.N., Yu W.T.S., Lim C.E.D. (2025). Integrative gut health: How fermented foods bridge ancient Eastern wisdom and modern microbiome science. J. Tradit. Chin. Med. Sci..

[37] Olson S., Welton L., Jahansouz C. (2024). Perioperative considerations for the surgical treatment of Crohn’s disease with discussion on surgical antibiotics practices and impact on the gut microbiome. Antibiotics.

[38] Li Y., Chen L., Xing C., Ding C., Zhang H., Wang S., Long Y., Guo J., Liao Q., Zhang T. (2021). Changes in serum lactate level predict postoperative intra-abdominal infection after pancreatic resection. World J. Surg..

[39] Cheong C.M., Golder A.M., Horgan P.G., Roxburgh C.S.D., McMillan D.C. (2022). Relationship between pre-operative glycated haemoglobin and surgical site infection in patients undergoing elective colon cancer surgery. Oncol. Lett..

[40] Liu S., Miao J., Wang G., Wang M., Wu X., Guo K., Feng M., Guan W., Ren J. (2017). Risk factors for postoperative surgical site infections in patients with Crohn’s disease receiving definitive bowel resection. Sci. Rep..

[41] Liu L., Liu L., Liang L.C., Zhu Z.Q., Wan X., Dai H.B., Huang Q. (2018). Impact of preoperative anemia on perioperative outcomes in patients undergoing elective colorectal surgery. Gastroenterol. Res. Pract..

[42] Čopi Jerman A., Zver Skomina J., Lučovnik M., Jeverica S. (2025). Effect of universal antibiotic prophylaxis on prevalence of surgical site infection after cesarean section. J. Clin. Med..

