# Supplementary material for: Perioperative Intravenous Antibiotic Regimens and Surgical Site Infection After Colorectal Surgery: Clinical Associations and External Evaluation of Risk Prediction Models
Source: Infect Dis Rep. 2026 Oct 6;18(5):113. doi: 10.3390/idr18050113 (PMC13704396; doi:10.3390/idr18050113)
Supplement: Supplementary file 1 [file idr-18-00113-s001.zip › idr-4532474-supplementary.pdf]

Supplementary Materials

Univariable Screening

Candidate covariates were selected at the initial screening stage based on a combination of univariable association with postoperative SSI and clinical relevance as potential confounders. Variables with $P<0.05$ in univariable logistic regression were retained, while clinically relevant covariates were also considered irrespective of univariable statistical significance. A change of at least 10% in any antibiotic-regimen odds ratio when a covariate was added individually to the exposure-only model was used as an additional supporting criterion. Accordingly, 24 covariates were considered in the subsequent multivariable analysis, with the perioperative intravenous antibiotic regimen forced into all models.

Supplementary Table S1. Univariable associations between candidate covariates and postoperative surgical site infection.

**Variable**	**OR**	**95% Confidence Interval**	**P value**
**Demographic characteristics**			
Sex: Male vs Female	1.075	1.007–1.147	0.029
Age, per 1-year increase	1.002	0.991–1.014	0.683
**Comorbidities and clinical history**			
Immunosuppression: Yes vs No	1.050	0.523–2.110	0.891
Inflammatory bowel disease: Yes vs No	1.398	0.830–2.353	0.207
Malnutrition within the previous year: Yes vs No	0.570	0.237–1.369	0.208
Malignancy: Yes vs No	0.800	0.490–1.307	0.373
Coagulopathy: Yes vs No	0.718	0.295–1.748	0.466
Chronic kidney disease: Yes vs No	1.160	0.594–2.264	0.664
Tobacco use within the previous year: Yes vs No	1.081	0.692–1.687	0.733
Diabetes mellitus: Yes vs No	0.920	0.477–1.772	0.802
**Previous healthcare utilisation and infection history**			
Hospitalisation within the previous year: Yes vs No	0.818	0.567–1.179	0.281
Intra-abdominal infection within the previous year: Yes vs No	1.264	0.699–2.285	0.438
**Preoperative haematological parameters**			
Haemoglobin, per 1-unit increase	0.888	0.817–0.965	0.005
Red blood cell count, per 1-unit increase	0.725	0.572–0.917	0.007
Haematocrit, per 1-unit increase	0.962	0.935–0.990	0.008
White blood cell count, per 1-unit increase	1.044	0.994–1.097	0.082
Platelet count, per 1-unit increase	1.001	0.999–1.003	0.446
**Preoperative renal function and glucose parameters**			
Serum creatinine, per 1-unit increase	1.262	0.879–1.812	0.194
Blood urea nitrogen, per 1-unit increase	1.029	1.006–1.053	0.014

Blood glucose, per 1-unit increase	1.007	1.000–1.014	0.045
**Preoperative electrolyte and mineral parameters**			
Serum potassium, per 1-unit increase	1.362	0.774–2.397	0.258
Serum chloride, per 1-unit increase	0.977	0.917–1.041	0.437
Total serum calcium, per 1-unit increase	0.629	0.454–0.872	0.006
Serum magnesium, per 1-unit increase	1.764	0.756–4.119	0.181
Serum phosphate, per 1-unit increase	1.222	0.968–1.544	0.092
**Preoperative biochemical and acid–base parameters**			
Serum sodium, per 1-unit increase	0.930	0.885–0.978	0.005
Lactate, per 1-unit increase	1.203	1.058–1.367	0.005
Total carbon dioxide, per 1-unit increase	0.945	0.907–0.983	0.006
Base excess, per 1-unit increase	0.966	0.940–0.991	0.009
Bicarbonate, per 1-unit increase	0.940	0.897–0.985	0.010
**Preoperative urinalysis parameters**			
Urinary red blood cells, per 1-unit increase	0.985	0.975–0.996	0.007
Urinary protein, per 1-unit increase	0.998	0.996–0.999	0.008

Supplementary Table S2. Multicollinearity assessment of candidate covariates.

Variable	VIF
Age	1.213
Sex	1.125
Hospitalisation within the previous year	1.946
Intra-abdominal infection within the previous year	1.082
Inflammatory bowel disease	1.085
Immunosuppression	1.089
Diabetes mellitus	1.114
Lactate	1.262
Blood glucose	1.137
Serum sodium	2.277
Total serum calcium	1.308
Chronic kidney disease	1.123
Blood urea nitrogen	1.435
Urinary red blood cells	1.075
Urinary protein	1.080
Haemoglobin	1.147
Haematocrit	1.078
Total carbon dioxide	1.127
Base excess	1.088
Bicarbonate	1.609
Tobacco use within the previous year	1.073

Malnutrition within the previous year	1.078
Red blood cell count	1.224
Malignancy	1.115

Model Selection

The perioperative intravenous antibiotic regimen was forced into all six models. Model 1 adjusted for age, sex, hospitalisation within the previous year and intra-abdominal infection within the previous year. Model 2 additionally included inflammatory bowel disease, immunosuppression and diabetes mellitus. Model 3 further added lactate, blood glucose, serum sodium and total serum calcium. Model 4 additionally incorporated chronic kidney disease, blood urea nitrogen, urinary red blood cells and urinary protein. Model 5 further included haemoglobin, haematocrit, total carbon dioxide and base excess. Model 6 additionally included bicarbonate, tobacco use within the previous year, malnutrition within the previous year, red blood cell count and malignancy.

Model fit improved progressively from Model 1 to Model 5, with the log likelihood increasing from −362.151 to −314.847 and the AIC decreasing from 740.302 to 677.695. Model 5 significantly improved fit over Model 4 (likelihood-ratio test, $P<0.001$) and had the lowest AIC. Adding the remaining covariates in Model 6 did not significantly improve model fit ($P=0.253$) and increased the AIC to 681.107; therefore, Model 5 was selected as the primary model. The association estimate for Group 3 versus Group 1 remained broadly consistent across all models, with the 95% confidence interval including unity in Model 5. In Model 5, VIF values ranged from 1.035 to 1.166, all substantially below the prespecified threshold of 15, indicating no concerning multicollinearity among the included covariates.

Supplementary Table S3. Comparison of sequential multivariable logistic regression models for postoperative surgical site infection.

Model	Log likelihood	AIC	BIC	LR-test P
Model 1	-362.151	740.302	777.068	—
Model 2	-356.205	734.411	784.964	0.0078
Model 3	-338.726	707.452	776.389	< 0.0001
Model 4	-329.080	696.160	783.479	0.0007
Model 5	-314.847	677.695	787.994	< 0.0001
Model 6	-311.553	681.107	814.384	0.2531

Supplementary Table S4. Multicollinearity assessment of covariates.

Variable	VIF
Age	1.123
Sex	1.054
Hospitalisation within the previous year	1.064

Intra-abdominal infection within the previous year	1.048
Inflammatory bowel disease	1.036
Immunosuppression	1.035
Diabetes mellitus	1.056
Lactate	1.104
Blood glucose	1.080
Serum sodium	1.046
Total serum calcium	1.166
Chronic kidney disease	1.043
Blood urea nitrogen	1.133
Urinary red blood cells	1.061
Urinary protein	1.053
Haemoglobin	1.037
Haematocrit	1.052
Total carbon dioxide	1.086
Base excess	1.045

Sensitivity analysis

Exploratory interaction signals for inflammatory bowel disease were observed across the examined sensitivity specifications, with global interaction P values ranging from 0.012 to 0.042. Compared with Group 1, the relative association for Group 2 was consistently stronger among patients with inflammatory bowel disease, with RORs ranging from 1.42 to 2.02 and all 95% confidence intervals excluding 1. The interaction estimates for Group 3 versus Group 1 were consistently below 1, reaching statistical significance in the reference sensitivity specification and weaker-penalty analysis, whereas no significant interaction was observed for Group 4 versus Group 1. The interaction between previous intra-abdominal infection and the Group 2 versus Group 1 comparison remained directionally consistent across all sensitivity analyses, with RORs ranging from 0.48 to 0.80 and all 95% confidence intervals excluding 1. In contrast, the interaction estimates for Group 3 and Group 4 versus Group 1 were not statistically significant. However, the global interaction tests did not reach statistical significance across the alternative model specifications and penalty strengths, with P values ranging from 0.059 to 0.100, indicating insufficient evidence that previous intra-abdominal infection modified the regimen–SSI association overall. All subgroup findings remain hypothesis-generating because of small subgroup sizes, multiple testing and residual confounding; they do not support subgroup-directed escalation of prophylaxis.

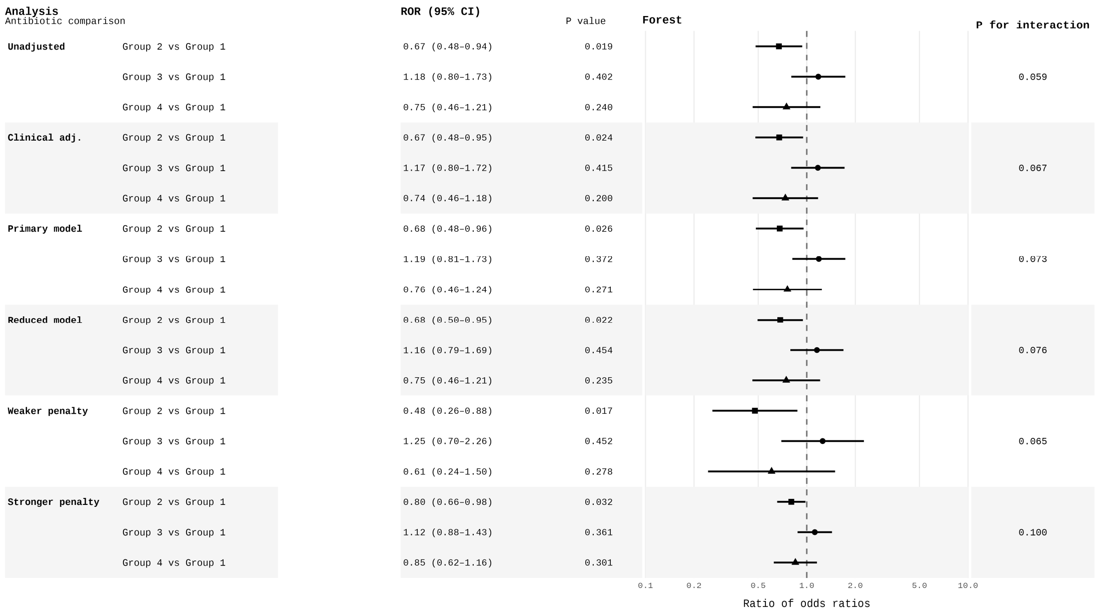

Supplementary Figure S1. Sensitivity analyses of effect modification by previous intra-abdominal infection on the association between perioperative antibiotic regimen and surgical site infection. The analyses shown in this figure represent alternative sensitivity specifications and are distinct from the primary Model 5 interaction analysis reported in the main manuscript, for which the global interaction P value was 0.030. The row labelled “Primary model” refers to the reference

specification within this sensitivity-analysis framework and should not be interpreted as the primary Model 5 interaction analysis reported in Figure 2.

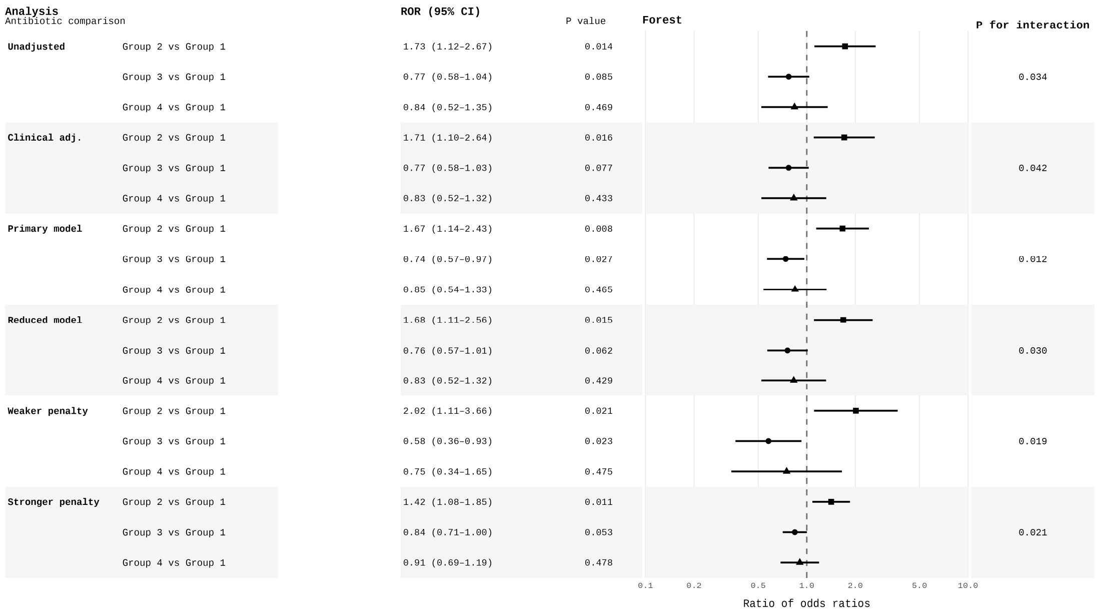

Supplementary Figure S2. Sensitivity analyses of effect modification by inflammatory bowel disease on the association between perioperative antibiotic regimen and surgical site infection. The analyses shown in this figure represent alternative sensitivity specifications and are distinct from the primary Model 5 interaction analysis reported in the main manuscript, for which the global interaction P value was 0.050. The row labelled “Primary model” refers to the reference specification within this sensitivity-analysis framework and should not be interpreted as the primary Model 5 interaction analysis reported in Figure 2.

Machine learning

Candidate predictors were initially evaluated using LASSO logistic regression, with the regularisation parameter selected by cross-validation (C=0.274). Supplementary Figure S3 presents the coefficient trajectories of the candidate variables across the regularisation path. LASSO was used as an initial screening procedure, and the screened variables were subsequently refined according to clinical relevance, routine perioperative availability and consistency of availability across the development and external datasets. This process yielded a fixed final predictor set comprising age, blood glucose, serum sodium, total serum calcium, blood urea nitrogen, haemoglobin, total carbon dioxide, base excess, lactate and perioperative antibiotic group. SSI was specified as the outcome and was therefore excluded from the predictor set. The same final predictor set was used consistently to develop and evaluate all six machine-learning models.

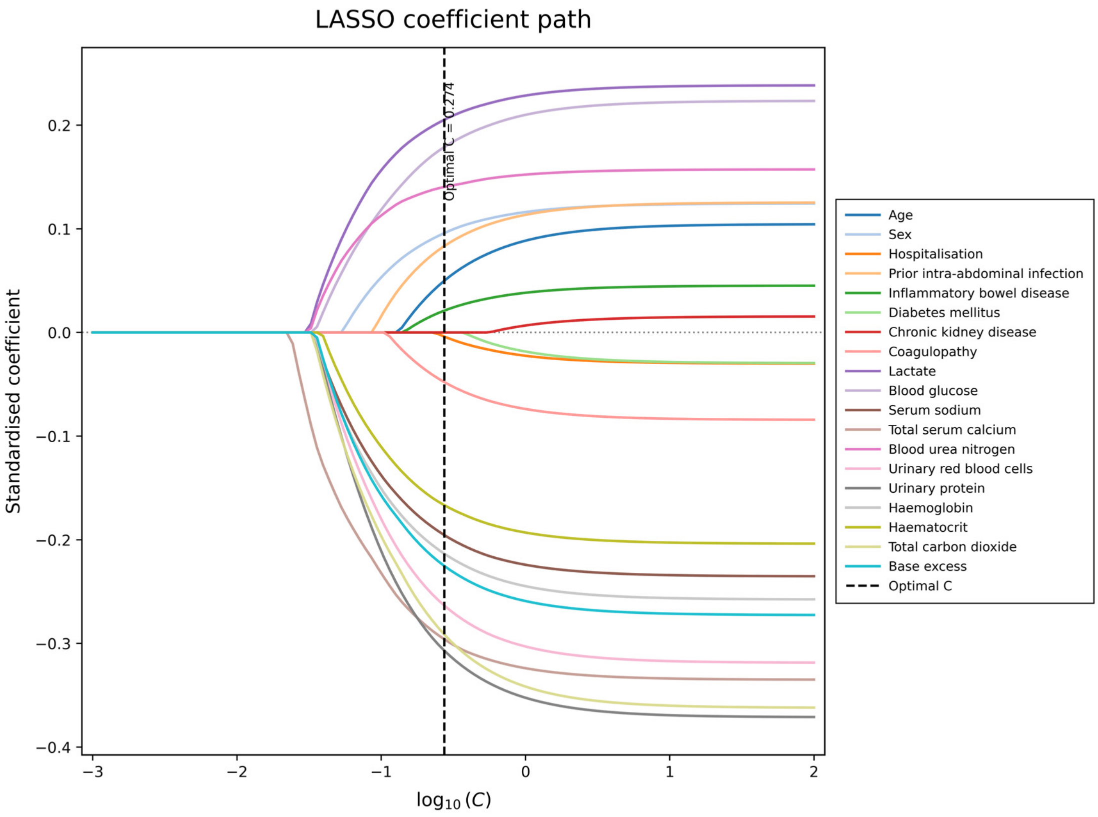

Supplementary Figure S3. LASSO coefficient paths and selection of the optimal regularisation parameter. Coefficient trajectories are shown for the candidate predictors displayed during the initial screening stage. The final predictor set was subsequently determined using combined statistical and clinical criteria.

Supplementary Table S5. Variable dictionary for machine learning model development.

Variable name	Variable type	Description
Age	Continuous	Patient age at the index admission, measured in years.
Blood_glucose	Continuous	Preoperative blood glucose concentration, measured in mg/dL.
Serum_sodium	Continuous	Preoperative serum sodium concentration, measured in mmol/L.
Total_serum_calcium	Continuous	Preoperative total serum calcium concentration, measured in mg/dL.
Blood_urea_nitrogen	Continuous	Preoperative blood urea nitrogen concentration, measured in mg/dL.
Haemoglobin	Continuous	Preoperative haemoglobin concentration, measured in g/dL.
Total_carbon_dioxide	Continuous	Preoperative total carbon dioxide concentration, measured in mmol/L.
Base_excess	Continuous	Preoperative base excess, reflecting the metabolic component of acid–base balance, measured in mmol/L.
Lactate	Continuous	Preoperative blood lactate concentration, measured in mmol/L.

SSI	Binary	Postoperative surgical site infection: 0 = no SSI and 1 = SSI.
Antibiotic_group	Categorical	Perioperative intravenous antibiotic regimen: Group 1 = fluoroquinolone–metronidazole combination; Group 2 = cephalosporin–metronidazole combination; Group 3 = β-lactam regimen with intrinsic aerobic and anaerobic activity; Group 4 = alternative perioperative intravenous antibiotic regimen.

Supplementary Table S6. Model parameter table.

**Model**	**Parameter**	**Setting**
Logistic regression	Standardisation	StandardScaler()
Logistic regression	Penalty	L2
Logistic regression	C	1
Logistic regression	Class weight	balanced
Logistic regression	Solver	lbfgs
Logistic regression	Maximum iterations	3000
Logistic regression	Random seed	42
Logistic regression	Classification threshold	0.596
Random forest	Number of trees	350
Random forest	Maximum tree depth	5
Random forest	Minimum samples per leaf	5
Random forest	Maximum features	sqrt
Random forest	Class weight	balanced
Random forest	Split criterion	gini
Random forest	Bootstrap sampling	TRUE
Random forest	Random seed	42
Random forest	Classification threshold	0.312
KNN	Standardisation	StandardScaler()
KNN	Number of neighbours	21
KNN	Weighting method	distance
KNN	Distance metric	Euclidean distance
KNN	Minkowski parameter p	2
KNN	Search algorithm	auto
KNN	Classification threshold	0.5
XGBoost	Number of estimators	250
XGBoost	Maximum tree depth	3
XGBoost	Learning rate	0.035
XGBoost	Subsample ratio	0.85
XGBoost	Feature sampling ratio	0.85
XGBoost	Minimum child weight	5
XGBoost	L2 regularisation lambda	2
XGBoost	L1 regularisation alpha	0.2

XGBoost	Evaluation metric	logloss
XGBoost	Random seed	42
XGBoost	Classification threshold	0.523
CatBoost	Number of iterations	300
CatBoost	Tree depth	4
CatBoost	Learning rate	0.035
CatBoost	L2 leaf regularisation	5
CatBoost	Loss function	Logloss
CatBoost	Random seed	42
CatBoost	Verbose output	FALSE
CatBoost	Allow writing files	FALSE
CatBoost	Classification threshold	0.498
LightGBM	Number of estimators	250
LightGBM	Maximum tree depth	3
LightGBM	Number of leaves	7
LightGBM	Learning rate	0.035
LightGBM	Minimum child samples	20
LightGBM	Subsample ratio	0.85
LightGBM	Feature sampling ratio	0.85
LightGBM	L2 regularisation lambda	2
LightGBM	L1 regularisation alpha	0.2
LightGBM	Random seed	42
LightGBM	Verbosity	-1
LightGBM	Classification threshold	0.447

In the MIMIC-IV internal validation cohort, ROC-AUC values ranged from 0.904 for XGBoost to 0.922 for logistic regression; KNN achieved 0.921 (Supplementary Figure S4). Precision–recall performance was similarly high, with PR-AUC values of 0.803–0.819, substantially exceeding the SSI prevalence of 0.200 (Supplementary Figure S5). In the independent external evaluation cohort, logistic regression had the numerically highest PR-AUC at 0.560 (95% CI 0.402–0.743), followed by KNN at 0.521 (95% CI 0.372–0.727), although all models showed lower precision–recall performance than in internal validation (Supplementary Figure S6). KNN demonstrated the most favourable calibration in both cohorts, with Brier scores of 0.082 and 0.107 and expected calibration errors of 0.039 and 0.067 in the internal validation and external evaluation cohorts, respectively; logistic regression showed comparatively poorer calibration, particularly in the external cohort (Brier score 0.197; expected calibration error 0.236) (Supplementary Figures S7 and S8). At the validation-derived threshold of 0.596, the logistic regression model correctly classified 149 of 176 non-SSI patients and 37 of 44 SSI patients, corresponding to a specificity of 84.7% and sensitivity of 84.1% (Supplementary Figure S9).

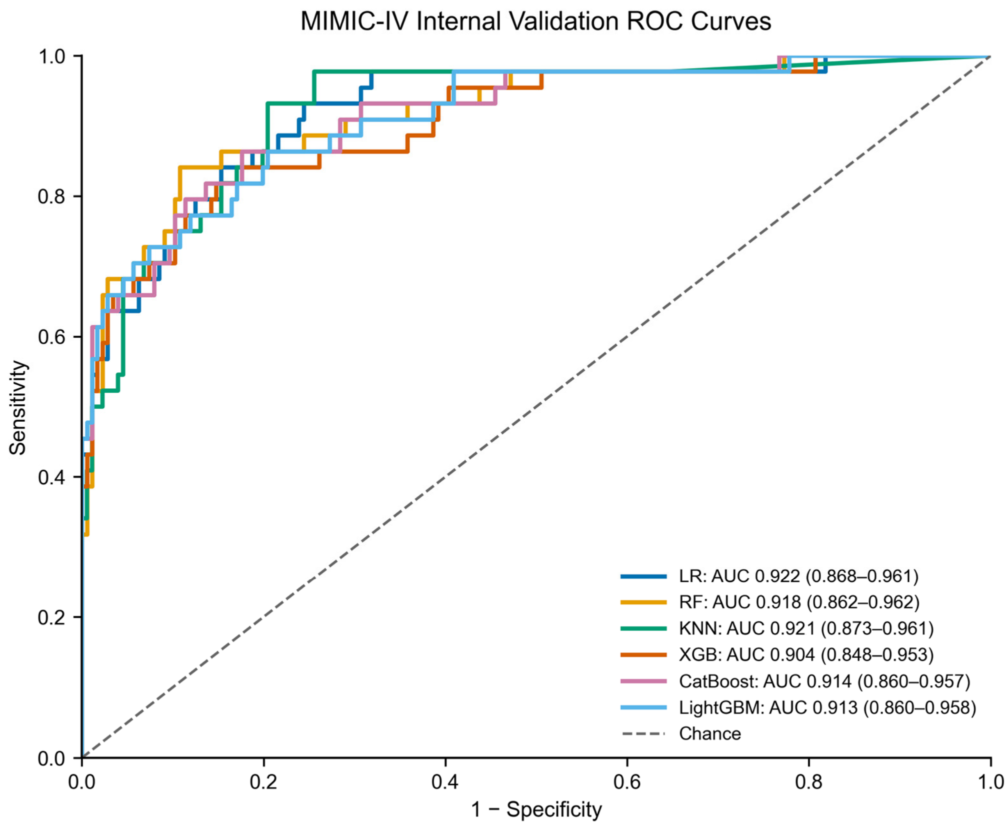

Supplementary Figure S4. Receiver operating characteristic curves of six models in the MIMIC-IV internal validation cohort.

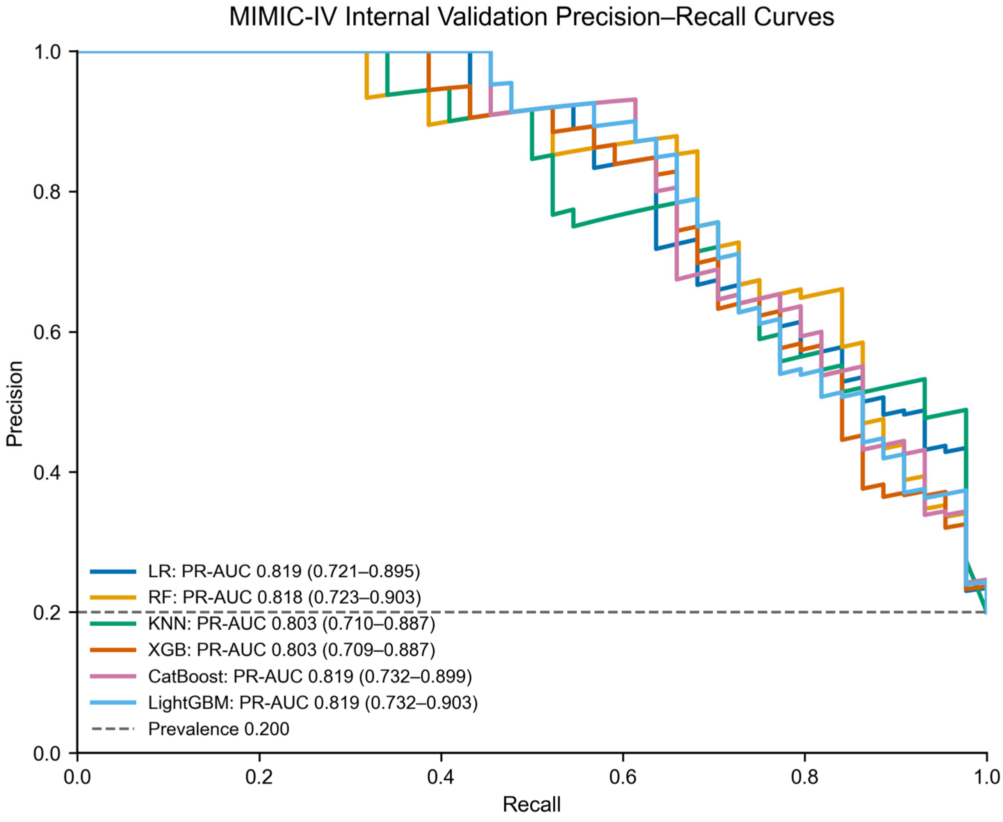

Supplementary Figure S5. Precision–recall curves of six models in the MIMIC-IV internal validation cohort.

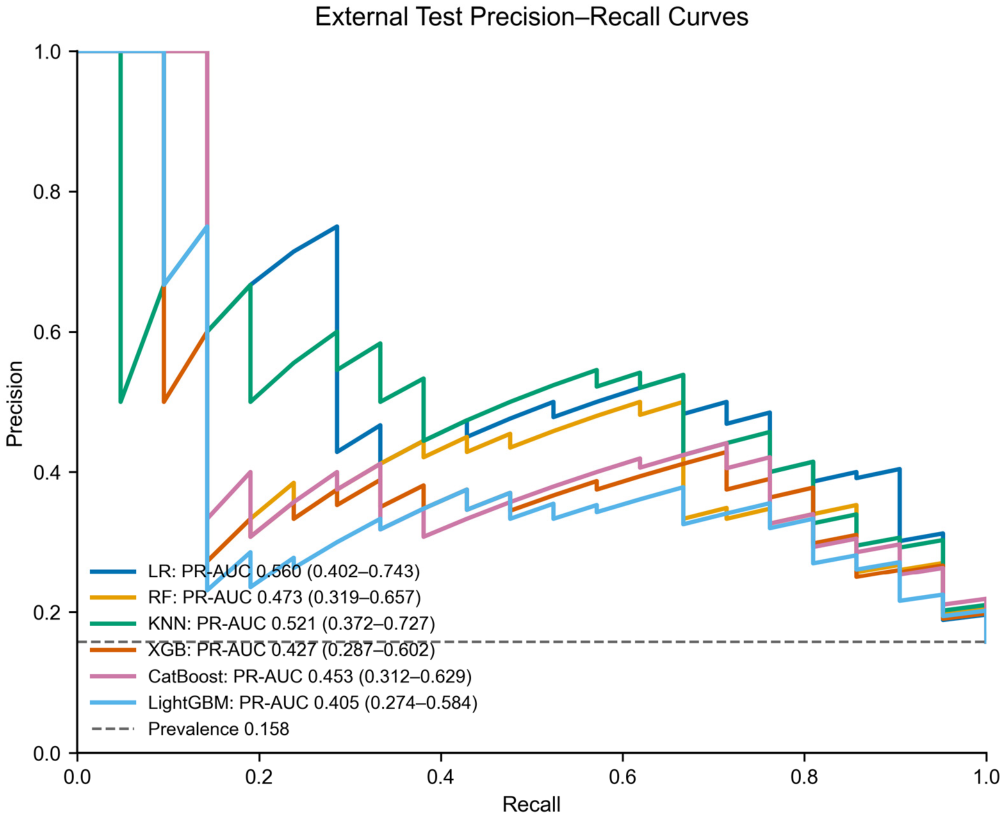

Supplementary Figure S6. Precision–recall curves of six models in the independent external evaluation cohort.

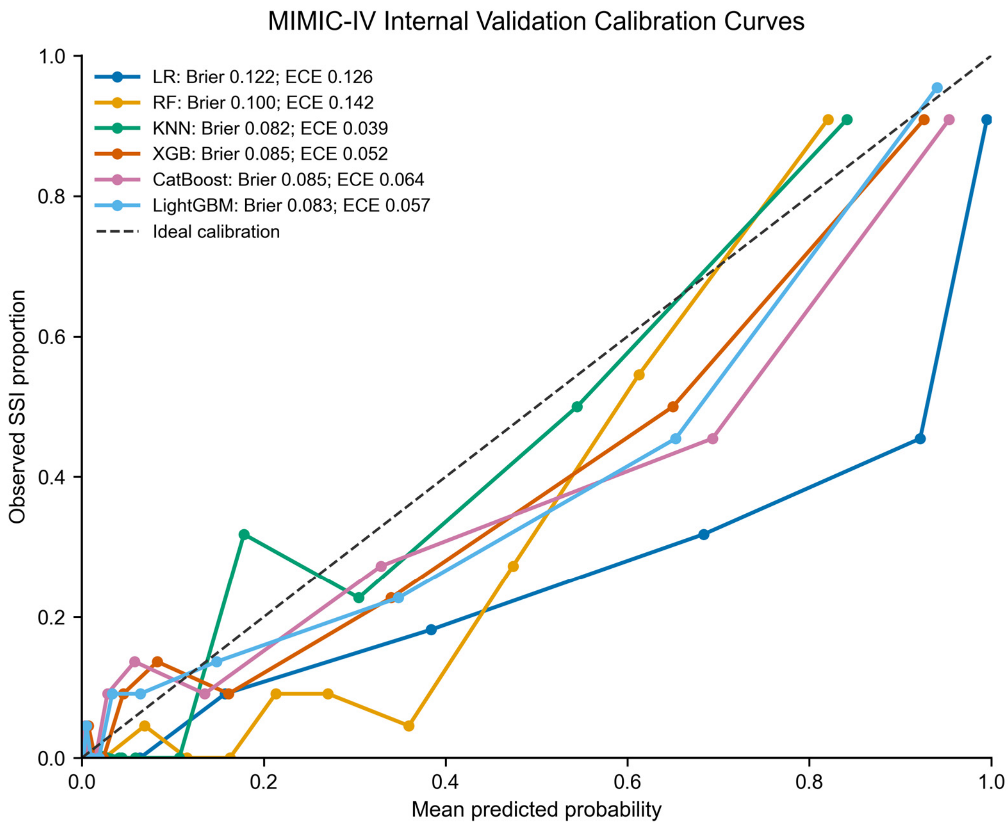

Supplementary Figure S7. Calibration curves of six models in the MIMIC-IV internal validation cohort.

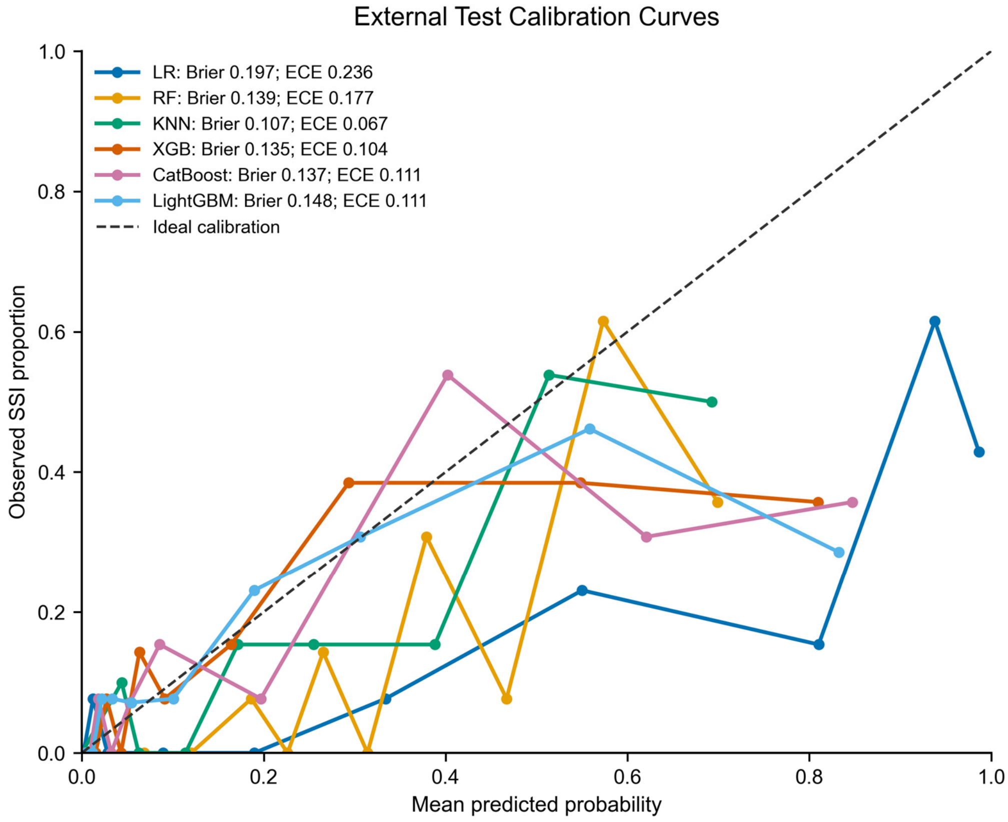

Supplementary Figure S8. Calibration curves of six models in the independent external evaluation cohort.

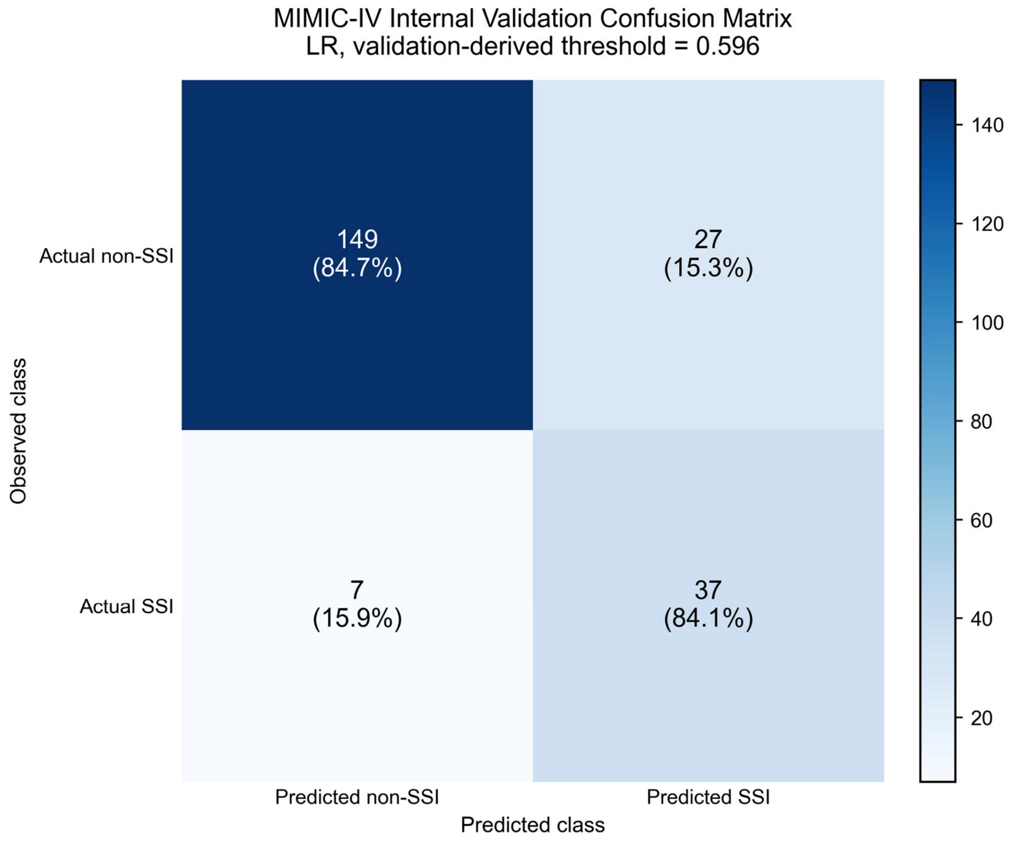

Supplementary Figure S9. Confusion matrix of the logistic regression model in the MIMIC-IV internal validation cohort at the validation-derived threshold.

Supplementary Table S7. Baseline characteristics of the training, internal validation, and external evaluation cohorts.

Characteristic	Training cohort (N=512)	Validation cohort (N=220)	External evaluation cohort (N=133)	P value
Age, years	62.0 [50.0, 72.0]	65.0 [53.0, 71.0]	71.0 [62.0, 82.0]	<0.001
Blood glucose, mg/dL	120.0 [93.0, 146.2]	123.0 [99.8, 152.0]	152.0 [122.0, 190.0]	<0.001
Serum sodium, mmol/L	138.8 [136.2, 141.1]	139.1 [136.3, 141.1]	138.0 [136.0, 141.0]	0.717
Total serum calcium, mg/dL	8.7 [8.2, 9.1]	8.6 [8.2, 9.0]	7.9 [7.5, 8.4]	<0.001
Blood urea nitrogen, mg/dL	17.0 [10.0, 24.0]	19.0 [12.8, 25.2]	14.0 [9.0, 18.0]	<0.001
Haemoglobin, g/dL	11.0 [9.3, 12.3]	10.6 [9.1, 12.1]	10.5 [9.3, 11.7]	0.129

Total carbon dioxide, mmol/L	22.8 [19.0, 25.3]	21.9 [18.9, 25.2]	23.0 [21.0, 25.0]	0.065
Base excess, mmol/L	−3.1 [−7.6, 1.5]	−2.2 [−7.7, 2.1]	−3.0 [−7.0, 1.2]	0.773
Lactate, mmol/L	2.3 [1.4, 3.4]	2.2 [1.4, 3.1]	2.0 [1.1, 3.0]	0.081
Antibiotic group, n (%)				0.473
Group 1	157 (30.7%)	53 (24.1%)	42 (31.6%)	
Group 2	121 (23.6%)	59 (26.8%)	36 (27.1%)	
Group 3	145 (28.3%)	61 (27.7%)	33 (24.8%)	
Group 4	89 (17.4%)	47 (21.4%)	22 (16.5%)	
Surgical site infection, yes, n (%)	102 (19.9%)	44 (20.0%)	21 (15.8%)	0.536

Supplementary Table S8. Performance of six machine-learning models in the internal validation cohort.

Model	Accuracy	Recall	Specificity	Precision	F1-score	Brier-score	ECE
Logistic regression	0.845	0.841	0.847	0.578	0.685	0.122	0.126
Random forest	0.882	0.841	0.892	0.661	0.740	0.100	0.142
KNN	0.823	0.932	0.795	0.532	0.678	0.082	0.039
XGBoost	0.845	0.818	0.852	0.581	0.679	0.085	0.052
CatBoost	0.832	0.864	0.824	0.551	0.673	0.085	0.064
LightGBM	0.809	0.864	0.795	0.514	0.644	0.083	0.057

Supplementary Table S9. Distribution of major recorded perioperative intravenous regimens and grouped residual categories within each antibiotic exposure group in the MIMIC-IV cohort.

**Antibiotic group**	**Perioperative intravenous antibiotic regimen category**	**Patients, n (%)**
Group 1: Fluoroquinolone + metronidazole-based regimens	Ciprofloxacin + metronidazole	210 (100.0%)
Group 1 total		210 (100.0%)
Group 2: Cephalosporin + metronidazole-based regimens	Cefazolin + metronidazole	86 (47.8%)
	Ceftriaxone + metronidazole	82 (45.6%)
	Cefepime + metronidazole	12 (6.7%)
Group 2 total		180 (100.0%)

Group 3: β-lactam regimens with intrinsic aerobic and anaerobic activity	Piperacillin–tazobactam monotherapy	84 (40.8%)
	Piperacillin–tazobactam combination regimens	39 (18.9%)
	Non-piperacillin β-lactam regimens	83 (40.3%)
Group 3 total		206 (100.0%)
Group 4: Alternative perioperative intravenous regimens	Cephalosporin monotherapy	34 (25.0%)
	Metronidazole monotherapy	20 (14.7%)
	Fluoroquinolone monotherapy	11 (8.1%)
	Alternative single-agent or combination regimens	71 (52.2%)
Group 4 total		136 (100.0%)

Table note: Values are n (%) within each antibiotic exposure group. Some rows aggregate multiple recorded regimens; the table is not an exhaustive agent-level breakdown and does not imply clinical interchangeability.

Supplementary Table S10. Overall and subgroup-specific associations between perioperative intravenous antibiotic regimens and surgical site infection after colorectal surgery.

Variable	Level	Antibiotic group	Adjusted OR (95% CI)	P value	P for interaction
Perioperative antibiotic regimen	All patients	Group 1	Reference	—	—
		Group 2	1.05 (0.91–1.21)	0.493	
		Group 3	0.95 (0.84–1.08)	0.429	
		Group 4	0.95 (0.80–1.13)	0.585	
Age ≥65 years	<65 years	Group 1	Reference	—	0.166
		Group 2	1.18 (0.97–1.44)	0.102	
		Group 3	1.03 (0.87–1.21)	0.742	
		Group 4	0.96 (0.79–1.17)	0.687	
	≥65 years	Group 1	Reference	—	
		Group 2	0.90 (0.73–1.10)	0.310	
		Group 3	0.88 (0.71–1.09)	0.251	

		Group 4	0.94 (0.73–1.21)	0.632	
Sex	Male	Group 1	Reference	—	0.958
		Group 2	1.10 (0.91–1.34)	0.314	
		Group 3	0.98 (0.81–1.19)	0.854	
		Group 4	0.96 (0.78–1.19)	0.706	
	Female	Group 1	Reference	—	
		Group 2	0.99 (0.77–1.26)	0.911	
		Group 3	0.93 (0.76–1.14)	0.498	
		Group 4	0.95 (0.72–1.25)	0.717	
Hospitalisation within previous year	No	Group 1	Reference	—	0.088
		Group 2	1.16 (0.98–1.38)	0.088	
		Group 3	1.02 (0.84–1.23)	0.836	
		Group 4	0.84 (0.67–1.04)	0.115	
	Yes	Group 1	Reference	—	
		Group 2	0.92 (0.74–1.13)	0.413	
		Group 3	0.86 (0.71–1.05)	0.141	
		Group 4	1.08 (0.84–1.38)	0.557	
Intra-abdominal infection within previous year	No	Group 1	Reference	—	0.030
		Group 2	1.11 (0.95–1.30)	0.197	
		Group 3	0.93 (0.82–1.06)	0.288	
		Group 4	0.99 (0.85–1.16)	0.920	
	Yes	Group 1	Reference	—	
		Group 2	0.62 (0.40–0.96)	0.034	

		Group 3	1.02 (0.65–1.60)	0.932	
		Group 4	0.66 (0.40–1.09)	0.107	
Inflammatory bowel disease	No	Group 1	Reference	—	0.050
		Group 2	0.99 (0.86–1.13)	0.871	
		Group 3	1.01 (0.87–1.17)	0.923	
		Group 4	0.99 (0.82–1.18)	0.886	
	Yes	Group 1	Reference	—	
		Group 2	1.68 (1.12–2.50)	0.011	
		Group 3	0.64 (0.48–0.85)	0.002	
		Group 4	0.75 (0.47–1.19)	0.219	
Diabetes mellitus	No	Group 1	Reference	—	0.545
		Group 2	1.03 (0.87–1.20)	0.759	
		Group 3	0.96 (0.83–1.12)	0.625	
		Group 4	0.96 (0.81–1.13)	0.601	
	Yes	Group 1	Reference	—	
		Group 2	1.32 (0.84–2.07)	0.226	
		Group 3	0.88 (0.57–1.35)	0.548	
		Group 4	0.84 (0.43–1.61)	0.592	

Supplementary Table S11. Adjusted associations between perioperative intravenous antibiotic regimen groups and postoperative surgical site infection in model 5.

Comparison	Regression coefficient (β)	Standard error (SE)	Adjusted odds ratio (aOR)	95% confidence interval (95% CI)	P value
Group 2 vs Group 1	0.0488	0.0727	1.05	0.91–1.21	0.493

Group 3 vs Group 1	−0.0513	0.0641	0.95	0.84–1.08	0.429
Group 4 vs Group 1	−0.0513	0.0881	0.95	0.80–1.13	0.585
